# A deep dive into burn-mediated ARDS severity assessment: a retrospective study on hematological markers

**DOI:** 10.1038/s41598-024-62235-4

**Published:** 2024-06-05

**Authors:** Jeongsoo Park, Dohern Kym, Jun Hur, Jaechul Yoon, Myongjin Kim, Yong Suk Cho, Wook Chun, Dogeon Yoon

**Affiliations:** 1 Department of Surgery and Critical Care, Burn Center, Hangang Sacred Heart Hospital, Hallym University Medical Center, College of Medicine, Hallym University, 12, Beodeunaru-ro 7-gil, Yeongdeungpo-gu, Seoul, 07247 Korea; 2 Burn Institutes, Hangang Sacred Heart Hospital, Hallym University Medical Center, 12, Beodeunaru-ro 7-gil, Youngdeungpo-gu, Seoul, 07247 Korea

**Keywords:** ARDS, CBC, NLR, MPV, Platelet, Medical research, Risk factors

## Abstract

Acute Respiratory Distress Syndrome (ARDS) is a critical form of Acute Lung Injury (ALI), challenging clinical diagnosis and severity assessment. This study evaluates the potential utility of various hematological markers in burn-mediated ARDS, including Neutrophil-to-Lymphocyte Ratio (NLR), Mean Platelet Volume (MPV), MPV-to-Lymphocyte Ratio (MPVLR), Platelet count, and Platelet Distribution Width (PDW). Employing a retrospective analysis of data collected over 12 years, this study focuses on the relationship between these hematological markers and ARDS diagnosis and severity in hospitalized patients. The study establishes NLR as a reliable systemic inflammation marker associated with ARDS severity. Elevated MPV and MPVLR also emerged as significant markers correlating with adverse outcomes. These findings suggest these economical, routinely measured markers can enhance traditional clinical criteria, offering a more objective approach to ARDS diagnosis and severity assessment. Hematological markers such as NLR, MPV, MPVLR, Platelet count, and PDW could be invaluable in clinical settings for diagnosing and assessing ARDS severity. They offer a cost-effective, accessible means to improve diagnostic accuracy and patient stratification in ARDS. However, further prospective studies are necessary to confirm these findings and investigate their integration with other diagnostic tools in diverse clinical settings.

## Introduction

Acute Respiratory Distress Syndrome (ARDS) significantly impacts global health, particularly in burn patients. This life-threatening condition, marked by extensive lung inflammation, results in high morbidity and mortality^1^. The complexity of ARDS in burn patients underscores its criticality in medical research. Despite its severity, challenges in early detection and accurate prognosis persist, highlighting the need for ongoing research and innovation.

The pathophysiology of ARDS, especially concerning burn injuries, is intricate. A thorough understanding could lead to targeted therapeutic strategies, enhancing patient outcomes through more personalized and effective treatments^2^. Early detection of ARDS is vital for initiating timely and appropriate treatment, emphasizing the importance of this research.

Complete Blood Count (CBC) tests are a cornerstone of clinical diagnostics and have been widely used in healthcare for decades. CBC tests, a clinical diagnostic staple, offer extensive insights into a patient's blood cellular composition^3^. These tests are crucial for diagnosing and monitoring various diseases. Recent research suggests that certain CBC-derived ratio markers, like Neutrophil-to-Lymphocyte Ratio (NLR), Monocyte-to-Lymphocyte Ratio (MLR), Platelet-to-Lymphocyte Ratio (PLR), and Mean Platelet Volume-to-Platelet Ratio (MPVPR), might be valuable diagnostic and prognostic tools in various medical conditions, including ARDS^4–6^. These markers provide insights into the body’s inflammatory and immune responses, key to understanding ARDS pathogenesis.

Despite the known utility of these markers, their specific roles in ARDS precipitated by burn injuries remain underexplored, leaving a substantial gap in research. This study aims to fill this gap by assessing the utility of routine CBC tests as diagnostic tools for ARDS in burn patients—a potentially cost-effective approach that could make diagnostic processes more accessible and improve overall healthcare outcomes. By exploring the correlation between CBC-derived ratio markers and the severity of ARDS in burn patients, this research seeks to enhance the early detection and accurate prognosis of ARDS, thus contributing to more efficient and cost-effective healthcare solutions.

## Methods

### Study design and patient selection

This retrospective cohort study took place at the Burn Intensive Care Unit (BICU) of Hangang Sacred Heart Hospital. It involved 2757 adult patients aged 18 or older, admitted due to burn injuries from January 2010 through December 2022. All patients received daily CBC tests during their hospital stay. The study excluded patients under 18, and patients without burn injuries were intentionally excluded to ensure that the study findings directly address the distinct challenges and treatment needs of burn-induced ARDS patients, adhering to the Strengthening the Reporting of Observational Studies in Epidemiology (STROBE) guidelines.

### Data collection and outcome measures

Patient data for this study were extracted from the Clinical Data Warehouse (CDW), which had been prospectively collected throughout the patients' hospitalization in the Burn Intensive Care Unit (BICU). The data included essential demographic and clinical details such as gender, age, diagnosis, and length of stay.

**Figure 1:**
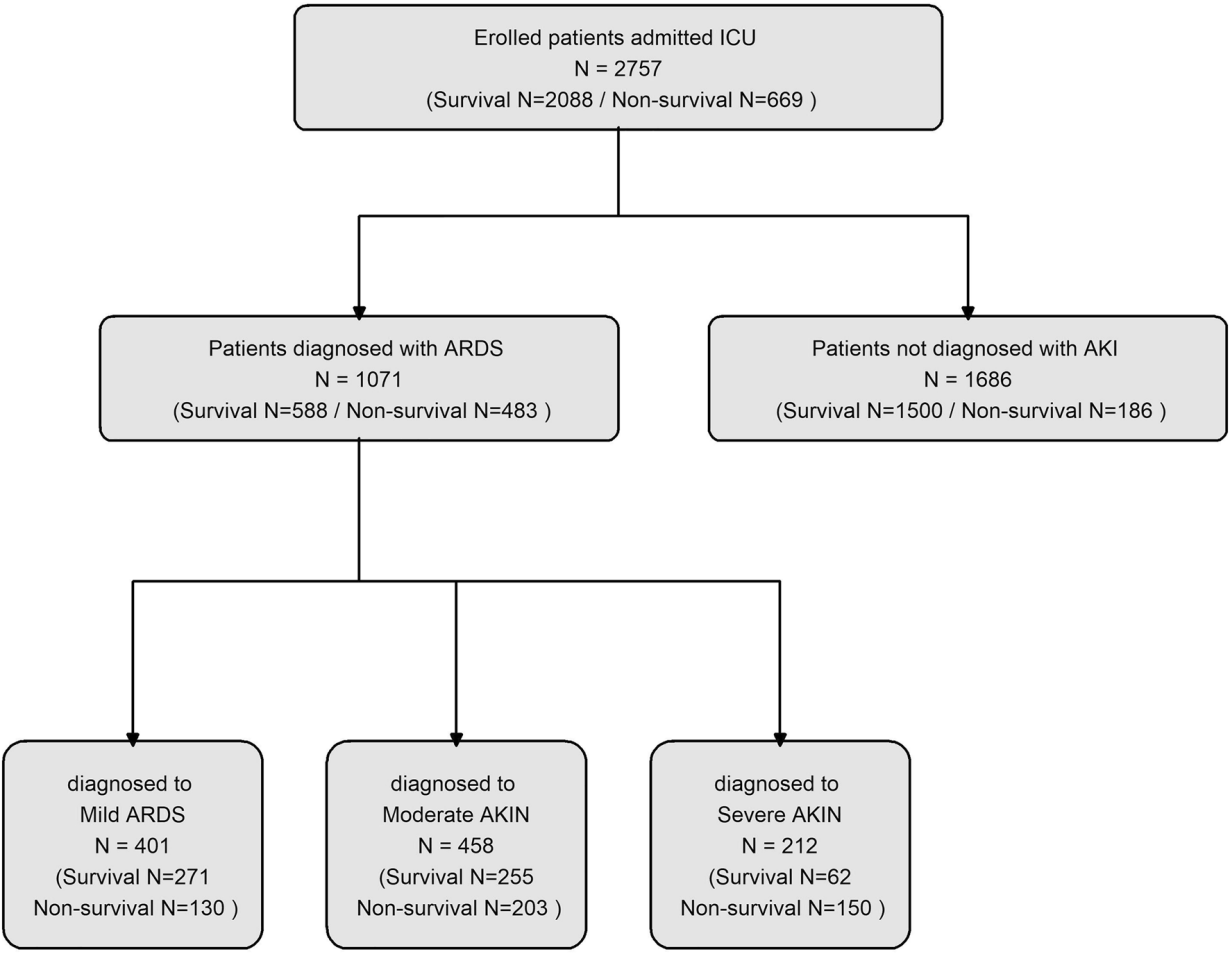
Flowchart depicting the enrollment process of patients in the study.

The diagnosis of ARDS was strictly based on the Berlin Definition^7^. A total of 1071 patients were identified as having developed ARDS during their hospitalization. To accurately depict the clinical process and patient categorization, Fig. 1 includes the ARDS grading and a flowchart of the included patients.

CBC parameters were routinely measured from the time of admission to BICU until discharge. These measurements were not restricted to the admission phase but were taken at regular intervals throughout each patient’s stay, meticulously covering all stages from the onset to the progression of ARDS. Importantly, the CBC data specifically utilized for analyzing the severity of ARDS were those collected at the precise time when ARDS severity was diagnosed.

Severity scores collected included APACHE IV, SOFA, ABSI^8^, rBaux^9^, and the Hangang Score^10^, which were developed and utilized by the hospital to assess the clinical severity of the patients. The primary objective of the study was to determine the incidence of ARDS, while the secondary outcome focused on the 60-day in-hospital mortality rate.

For the diagnosis of ARDS, it was essential to confirm that the observed respiratory failure was not influenced by cardiac conditions that could mimic ARDS, such as heart failure or fluid overload. Patients with pre-existing heart conditions were not excluded from the study; however, those whose respiratory symptoms could primarily be attributed to cardiac dysfunction were carefully differentiated. Echocardiographic data and comprehensive clinical histories were reviewed to ensure the respiratory distress observed was solely pulmonary in origin.

ARDS onset was confirmed if it occurred within a week of a known clinical insult or if there were new or worsening respiratory symptoms. Chest imaging was used to confirm bilateral opacities not fully explainable by effusions, lobular or lung collapse, or nodules. ARDS severity was assessed based on the ratio of arterial oxygenation to fractional inspired oxygen (FiO_2_), utilizing either positive end-expiratory pressure (PEEP) or continuous positive airway pressure (CPAP). Patients were classified as having mild (PaO_2_/FiO_2_ > 200 mmHg and ≤ 300 mmHg), moderate (PaO_2_/FiO_2_ > 100 mmHg and ≤ 200 mmHg), or severe ARDS (PaO_2_/FiO_2_ ≤ 100 mmHg).

Given the retrospective nature of the study and the approval from the Hangang Sacred Heart Hospital Institutional Review Board (IRB), the requirement for informed consent was waived. This approach ensured comprehensive data collection and analysis while adhering to ethical standards.

### Definition of ratio markers

1. Neutrophil-to-Lymphocyte Ratio (NLR): NLR is the ratio of the absolute neutrophil count to the absolute lymphocyte count (NLR = Absolute Neutrophil Count/Absolute Lymphocyte Count). Elevated NLR values are associated with adverse outcomes in various medical conditions^11^.
2. Platelet-to-Lymphocyte Ratio (PLR): PLR is determined by dividing the absolute platelet count by the absolute lymphocyte count (PLR = Absolute Platelet Count/Absolute Lymphocyte Count). High PLR, akin to NLR, indicates increased systemic inflammation and is linked to poorer prognoses in these diseases^12^.
3. Monocyte-to-Lymphocyte Ratio (MLR): MLR is calculated as the absolute monocyte count divided by the absolute lymphocyte count (MLR = Absolute Monocyte Count/Absolute Lymphocyte Count). An increased MLR is associated with worse prognoses in various diseases^12^.
4. Systemic Immune-Inflammation Index (SII): SII is computed by multiplying the platelet count by the neutrophil count and then dividing by the lymphocyte count (SII = (Platelet Count × Neutrophil Count)/Lymphocyte Count). Elevated SII values are correlated with poor postoperative conditions and prognoses in diverse diseases^13^.
5. Mean Platelet Volume-to-Platelet Ratio (MPVPR), Mean Platelet Volume-to-Lymphocyte Ratio (MPVLR), Mean Platelet Volume-to-Monocyte Ratio (MPVMR), Mean Platelet Volume-to-Neutrophil Ratio (MPVNR): These ratios involve dividing the mean platelet volume by the counts of platelets, lymphocytes, monocytes, and neutrophils, respectively (MPVPR = Mean Platelet Volume/Platelet Count; MPVLR = Mean Platelet Volume/Lymphocyte Count; MPVMR = Mean Platelet Volume/Monocyte Count; MPVNR = Mean Platelet Volume/Neutrophil Count). Though evidence is limited, some studies suggest the potential effectiveness of these markers in various conditions^14^.

### Data management and statistical analysis

Data management rigorously followed good clinical practice and data protection regulations. All patient identifiers were removed to maintain confidentiality. The data was thoroughly reviewed for completeness and consistency, with any discrepancies resolved by consulting the original documentation. To reduce the impact of outliers in the analysis, a technique known as winsorizing was utilized. Additionally, data scaling was performed to ensure uniformity across variables, preventing any single variable from disproportionately influencing the analysis.

Baseline demographic characteristics were reported as follows: for normally distributed continuous variables, means ± standard deviations were presented, while medians (25th–75th interquartile range) were used for non-normally distributed variables. The statistical tests employed to determine differences between groups depended on data normality, utilizing either the paired *t*-test or the Wilcoxon signed-rank test. Categorical variables were represented as percentages and analyzed using either the Chi-square or Fisher's exact tests.

To evaluate the diagnostic power of ARDS, regardless of severity, metrics like AUC, accuracy, sensitivity, specificity, positive predictive value, and negative predictive value were assessed. The Youden index was used to determine the optimal cutoff. The Vector Generalized Additive Model (VGAM) technique, suitable for analyzing unbalanced repeated measurement data, was used to predict ARDS severity, an ordinal outcome, with odds ratios derived using the VGAM package. The model was adjusted for age, Total Body Surface Area (TBSA), and inhalation injury, known significant predictors in burn patients. If this model did not predict mortality, Hazard Ratios (HR) were analyzed using Cox proportional hazard analysis. All statistical analyses were conducted using R software (Version 4.3.0).

### Ethics approval and consent to participate

The study was conducted in accordance with the Declaration of Helsinki and was approved by the Hangang Sacred Heart Hospital Institutional Review Board (IRB) (HG2022-011).

## Results

### Demographics and characteristics for enrolled patients

The study included 2758 patients, of whom 669 (24.3%) experienced mortality, and 1071 (38.8%) developed ARDS. Among the ARDS cases, the severity distribution was as follows: mild ARDS was observed in 401 patients, representing 37.4% of ARDS cases and 14.5% of the total study population; moderate ARDS was observed in 458 patients, accounting for 42.8% of ARDS cases and 16.8% of the total population; severe ARDS was diagnosed in 212 patients, constituting 19.8% of ARDS cases and 7.7% of the total patient cohort.

Notable differences in demographic and severity score variables were observed across different ARDS grades and between patients with and without ARDS, except for gender. The median age of participants was 52, with a majority (79.1%) being male. The median Total Body Surface Area (TBSA) affected by burns was 27%, and inhalation burns were present in 38.5% of patients. Higher severity scores, correlating with worse prognoses, were noted with increasing Acute Kidney Injury Network (AKIN) grades. Apart from Platelet-to-Lymphocyte Ratio (PLR) and Mean Platelet Volume-to-Monocyte Ratio (MPVMR), significant differences were observed in all CBC parameters and ratio markers when comparing ARDS grades and the occurrence of ARDS. Further details are provided in Table 1.

**Table 1:** Characteristics of enrolled patients by ARDS Berlin definition and ARDS status.

Group	Variables	ARDS Berlin definition						ARDS status		
		Overall, N = 2,757	No, N = 1686 (61.2%)	Mild, N = 401 (14.5%)	Moderate, N = 458 (16.6%)	Severe, N = 212 (7.69%)	p-value	Yes, N = 1071 (38.8%)	No, N = 1686 (61.2%)	p-value
Demographics	Mortality	669 (24.3%)	186 (11.0%)	130 (32.4%)	203 (44.3%)	150 (70.8%)	< 0.001	483 (45.1%)	186 (11.0%)	< 0.001
	Age						< 0.001			< 0.001
	Median [IQR]	52 [41, 63]	51 [40, 62]	52 [41, 62]	54 [43, 67]	54 [44, 64]		53 [42, 64]	51 [40, 62]	
	Sex						0.515			0.701
	Male	2180 (79.1%)	1329 (78.8%)	311 (77.6%)	365 (79.7%)	175 (82.5%)		851 (79.5%)	1,329 (78.8%)	
	Female	577 (20.9%)	357 (21.2%)	90 (22.4%)	93 (20.3%)	37 (17.5%)		220 (20.5%)	357 (21.2%)	
	Type						< 0.001			< 0.001
	FB	1918 (69.6%)	1067 (63.4%)	309 (77.1%)	360 (78.6%)	182 (85.8%)		851 (79.5%)	1067 (63.4%)	
	SB	275 (10.0%)	185 (11.0%)	42 (10.5%)	37 (8.1%)	11 (5.2%)		90 (8.4%)	185 (11.0%)	
	EB	369 (13.4%)	304 (18.1%)	27 (6.7%)	31 (6.8%)	7 (3.3%)		65 (6.1%)	304 (18.1%)	
	ChB	46 (1.7%)	28 (1.7%)	5 (1.2%)	10 (2.2%)	3 (1.4%)		18 (1.7%)	28 (1.7%)	
	CoB	147 (5.3%)	100 (5.9%)	18 (4.5%)	20 (4.4%)	9 (4.2%)		47 (4.4%)	100 (5.9%)	
	TBSA						< 0.001			< 0.001
	Median [IQR]	27 [15, 46]	21 [10, 33]	41 [26, 61]	40 [23, 62]	47 [30, 78]		42 [25, 63]	21 [10, 33]	
	Inhalation	1,063 (38.6%)	505 (30.0%)	189 (47.1%)	234 (51.1%)	135 (63.7%)	< 0.001	558 (52.1%)	505 (30.0%)	< 0.001
	LOS						< 0.001			< 0.001
	Median [IQR]	12 [5, 27]	7 [4, 17]	30 [18, 47]	25 [13, 41]	13 [5, 29]		25 [12, 43]	7 [4, 17]	
Severity Scores	Mortality	669 (24.3%)	186 (11.0%)	130 (32.4%)	203 (44.3%)	150 (70.8%)	< 0.001	483 (45.1%)	186 (11.0%)	< 0.001
	ABSI						< 0.001			< 0.001
	Median [IQR]	8 [6, 10]	7 [6, 8]	9 [8, 11]	9 [8, 12]	10 [8, 13]		9 [8, 12]	7 [6, 8]	
	rBaux						< 0.001			< 0.001
	Median [IQR]	88 [70, 110]	78 [64, 94]	104 [88, 122]	105 [89, 127]	113 [93, 139]		107 [90, 128]	78 [64, 94]	
	Hangang						< 0.001			< 0.001
	Median [IQR]	124 [112, 142]	116 [108, 126]	137 [126, 149]	140 [128, 155]	153 [134, 172]		140 [129, 157]	116 [108, 126]	
	APACHE_IV						< 0.001			< 0.001
	Median [IQR]	35 [23, 59]	28 [19, 43]	44 [32, 61]	50 [35, 68]	66 [49, 92]		50 [35, 69]	28 [19, 43]	
	SOFA						< 0.001			< 0.001
	Median [IQR]	3 [1, 4]	2 [0, 3]	3 [2, 5]	4 [3, 6]	5 [4, 8]		4 [3, 6]	2 [0, 3]	
WBC related	WBC						< 0.001			< 0.001
	Median [IQR]	11.1 [7.9, 17.7]	9.4 [7.1, 12.3]	17.2 [11.3, 23.9]	17.9 [10.5, 23.9]	20.4 [12.7, 23.9]		17.9 [11.2, 23.9]	9.4 [7.1, 12.3]	
	Neutrophil						< 0.001			< 0.001
	Median [IQR]	8.4 [5.6, 14.3]	6.8 [5.0, 9.6]	14.3 [9.1, 20.4]	14.6 [8.1, 20.4]	15.8 [9.4, 20.4]		14.5 [8.8, 20.4]	6.8 [5.0, 9.6]	
	Lymphocyte						< 0.001			< 0.001
	Median [IQR]	1.31 [0.92, 1.85]	1.30 [0.94, 1.70]	1.27 [0.90, 1.95]	1.32 [0.90, 2.23]	1.82 [1.00, 2.78]		1.36 [0.90, 2.35]	1.30 [0.94, 1.70]	
	Monocyte						< 0.001			< 0.001
	Median [IQR]	0.70 [0.50, 1.04]	0.64 [0.47, 0.89]	0.92 [0.51, 1.36]	0.83 [0.54, 1.27]	0.87 [0.50, 1.36]		0.88 [0.52, 1.33]	0.64 [0.47, 0.89]	
	Eosinophil						< 0.001			< 0.001
	Median [IQR]	0.14 [0.08, 0.25]	0.17 [0.10, 0.29]	0.10 [0.04, 0.20]	0.10 [0.05, 0.20]	0.10 [0.05, 0.20]		0.10 [0.05, 0.20]	0.17 [0.10, 0.29]	
	Basophil						< 0.001			< 0.001
	Median [IQR]	0.04 [0.03, 0.08]	0.04 [0.02, 0.07]	0.06 [0.03, 0.10]	0.06 [0.03, 0.10]	0.07 [0.04, 0.11]		0.06 [0.03, 0.10]	0.04 [0.02, 0.07]	
	Immature granulocyte						< 0.001			< 0.001
	Median [IQR]	0.09 [0.04, 0.25]	0.06 [0.03, 0.14]	0.19 [0.10, 0.41]	0.14 [0.07, 0.32]	0.30 [0.14, 1.05]		0.18 [0.09, 0.42]	0.06 [0.03, 0.14]	
RBC related	RBC						< 0.001			< 0.001
	Median [IQR]	3.98 [3.33, 4.86]	3.71 [3.24, 4.33]	4.72 [3.59, 5.20]	4.82 [3.70, 5.20]	5.03 [3.65, 5.20]		4.83 [3.64, 5.20]	3.71 [3.24, 4.33]	
	RDW						< 0.001			< 0.001
	Median [IQR]	13.20 [12.40, 14.32]	13.10 [12.24, 13.99]	13.40 [12.64, 14.40]	13.33 [12.64, 14.64]	13.75 [13.00, 15.75]		13.49 [12.64, 14.65]	13.10 [12.24, 13.99]	
	Hct						< 0.001			< 0.001
	Median [IQR]	37 [31, 46]	35 [30, 40]	45 [34, 46]	45 [35, 46]	46 [35, 46]		45 [35, 46]	35 [30, 40]	
	Hb						< 0.001			< 0.001
	Median [IQR]	12.20 [10.20, 15.10]	11.40 [9.90, 13.30]	14.90 [11.10, 16.20]	14.90 [11.40, 16.30]	15.20 [11.20, 16.40]		14.90 [11.20, 16.30]	11.40 [9.90, 13.30]	
	MCV						< 0.001			< 0.001
	Median [IQR]	91.7 [89.1, 94.9]	91.3 [88.8, 94.2]	92.1 [89.4, 95.9]	92.6 [89.7, 96.3]	93.1 [90.1, 96.2]		92.6 [89.6, 96.1]	91.3 [88.8, 94.2]	
	MCH						< 0.001			< 0.001
	Median [IQR]	30.70 [29.80, 31.80]	30.60 [29.70, 31.60]	30.90 [29.90, 32.20]	31.10 [30.00, 32.20]	31.00 [30.00, 32.10]		30.90 [30.00, 32.20]	30.60 [29.70, 31.60]	
	MCHC						< 0.001			< 0.001
	Median [IQR]	33.60 [32.80, 34.40]	33.50 [32.80, 34.30]	33.80 [32.90, 34.60]	33.75 [33.00, 34.50]	33.70 [32.70, 34.65]		33.80 [32.90, 34.60]	33.50 [32.80, 34.30]	
Platelet related	Platelet						< 0.001			< 0.001
	Median [IQR]	247 [171, 354]	253 [180, 368]	224 [152, 307]	233 [150, 310]	268 [175, 393]		241 [155, 329]	253 [180, 368]	
	MPV						< 0.001			< 0.001
	Median [IQR]	9.84 [9.24, 10.64]	9.76 [9.20, 10.39]	10.12 [9.49, 10.84]	10.04 [9.38, 11.00]	10.20 [9.45, 11.24]		10.12 [9.40, 11.00]	9.76 [9.20, 10.39]	
	PDW						< 0.001			< 0.001
	Median [IQR]	10.61 [9.48, 12.48]	10.10 [9.10, 11.56]	11.69 [10.30, 13.23]	11.65 [10.23, 14.00]	11.80 [10.20, 14.30]		11.70 [10.28, 13.78]	10.10 [9.10, 11.56]	
	PCT						< 0.001			< 0.001
	Median [IQR]	0.20 [0.15, 0.30]	0.20 [0.17, 0.30]	0.20 [0.10, 0.29]	0.20 [0.11, 0.30]	0.20 [0.17, 0.36]		0.20 [0.11, 0.30]	0.20 [0.17, 0.30]	
Ratios	NLR						< 0.001			< 0.001
	Median [IQR]	7 [4, 12]	6 [4, 8]	13 [8, 19]	12 [7, 20]	13 [7, 19]		12 [8, 19]	6 [4, 8]	
	PLR						0.541			0.235
	Median [IQR]	200 [133, 300]	203 [135, 304]	196 [132, 296]	192 [128, 297]	193 [126, 294]		193 [129, 296]	203 [135, 304]	
	MLR						< 0.001			< 0.001
	Median [IQR]	0.58 [0.39, 0.88]	0.51 [0.36, 0.72]	0.82 [0.53, 1.16]	0.75 [0.47, 1.16]	0.68 [0.43, 1.00]		0.76 [0.49, 1.14]	0.51 [0.36, 0.72]	
	SII						< 0.001			< 0.001
	Median [IQR]	1709 [941, 3,039]	1390 [817, 2,307]	2517 [1352, 4,163]	2559 [1306, 4143]	2587 [1364, 4479]		2569 [1334, 4223]	1390 [817, 2307]	
	MPVPR						< 0.001			< 0.001
	Median [IQR]	0.04 [0.03, 0.07]	0.04 [0.03, 0.06]	0.05 [0.04, 0.08]	0.05 [0.04, 0.09]	0.05 [0.03, 0.08]		0.05 [0.04, 0.09]	0.04 [0.03, 0.06]	
	MPVLR						< 0.001			< 0.001
	Median [IQR]	8 [6, 12]	8 [6, 11]	11 [7, 15]	10 [6, 16]	9 [6, 16]		10 [7, 16]	8 [6, 11]	
	MPVMR						0.009			0.558
	Median [IQR]	16 [11, 22]	16 [12, 22]	14 [9, 25]	15 [10, 25]	18 [11, 34]		15 [10, 27]	16 [12, 22]	
	MPVNR						< 0.001			< 0.001
	Median [IQR]	1.30 [0.84, 1.91]	1.46 [1.05, 2.03]	0.97 [0.60, 1.44]	0.92 [0.57, 1.73]	0.97 [0.63, 1.61]		0.95 [0.59, 1.63]	1.46 [1.05, 2.03]	

### Diagnostic performance of CBC parameters and ratios for predicting ARDS

**Figure 2:**
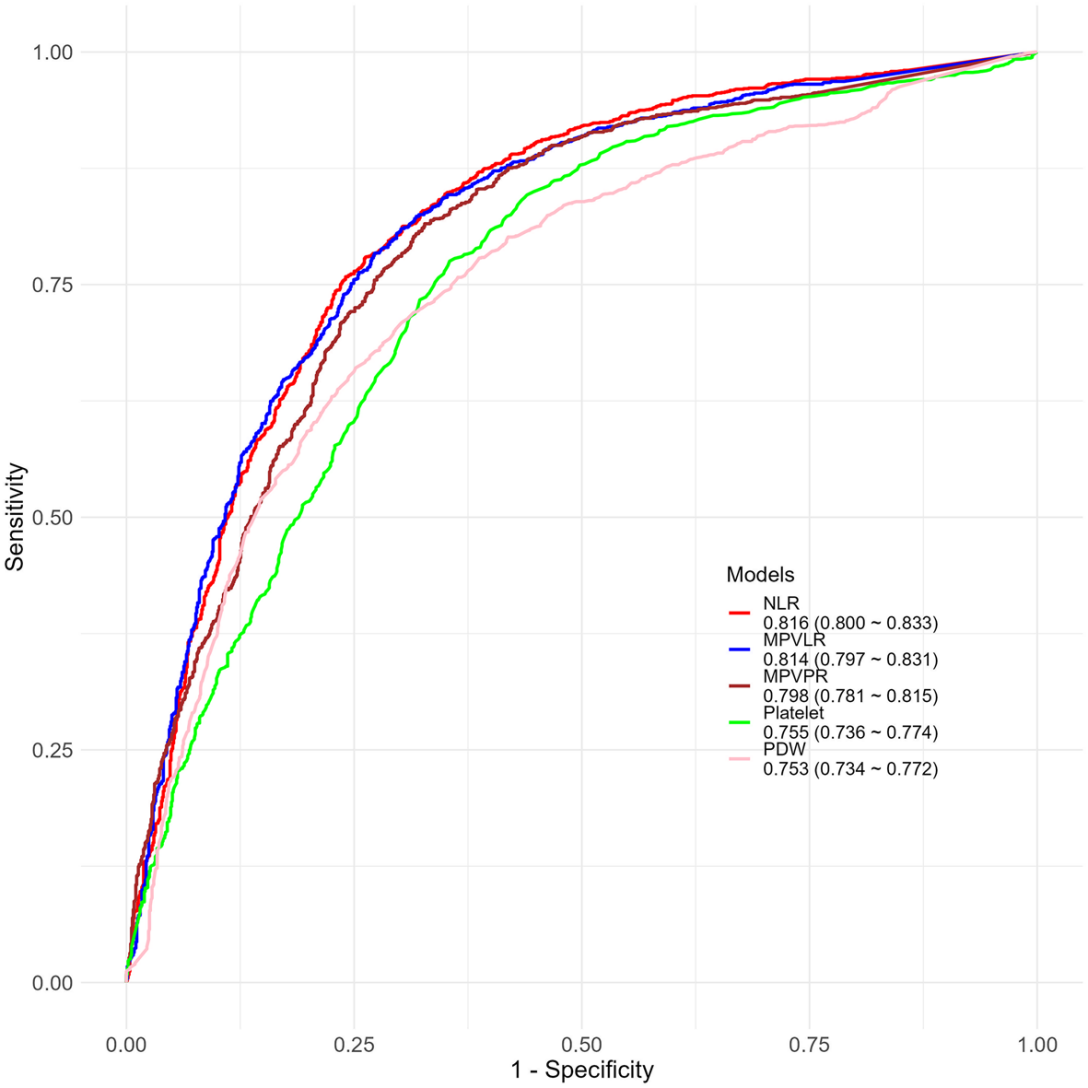
Top four AUC values for predicting ARDS.

In the study, WBC-related parameters, specifically immature granulocytes, exhibited an AUC value of 0.717 with a 95% CI ranging from 0.687 to 0.747. RBC-related parameters were analyzed, with RDW showing an AUC of 0.706 (CI: 0.687–0.726). For platelet-related parameters, both platelet count and PDW demonstrated AUC values above 0.7, specifically 0.755 (CI: 0.736–0.774) for platelet count and 0.753 (CI: 0.734–0.772) for PDW. Among the ratio markers, NLR, a WBC-related parameter, showed the highest AUC at 0.816 (CI: 0.800–0.833), followed by MPVLR and MPVPR with AUC values of 0.814 and 0.798, respectively. The AUC curves for the top five variables with the highest AUC values are presented in Fig. 2, and detailed AUC values and performance metrics for all variables can be found in Table S1.

### Severity discrimination power of ARDS grade

**Figure 3:**
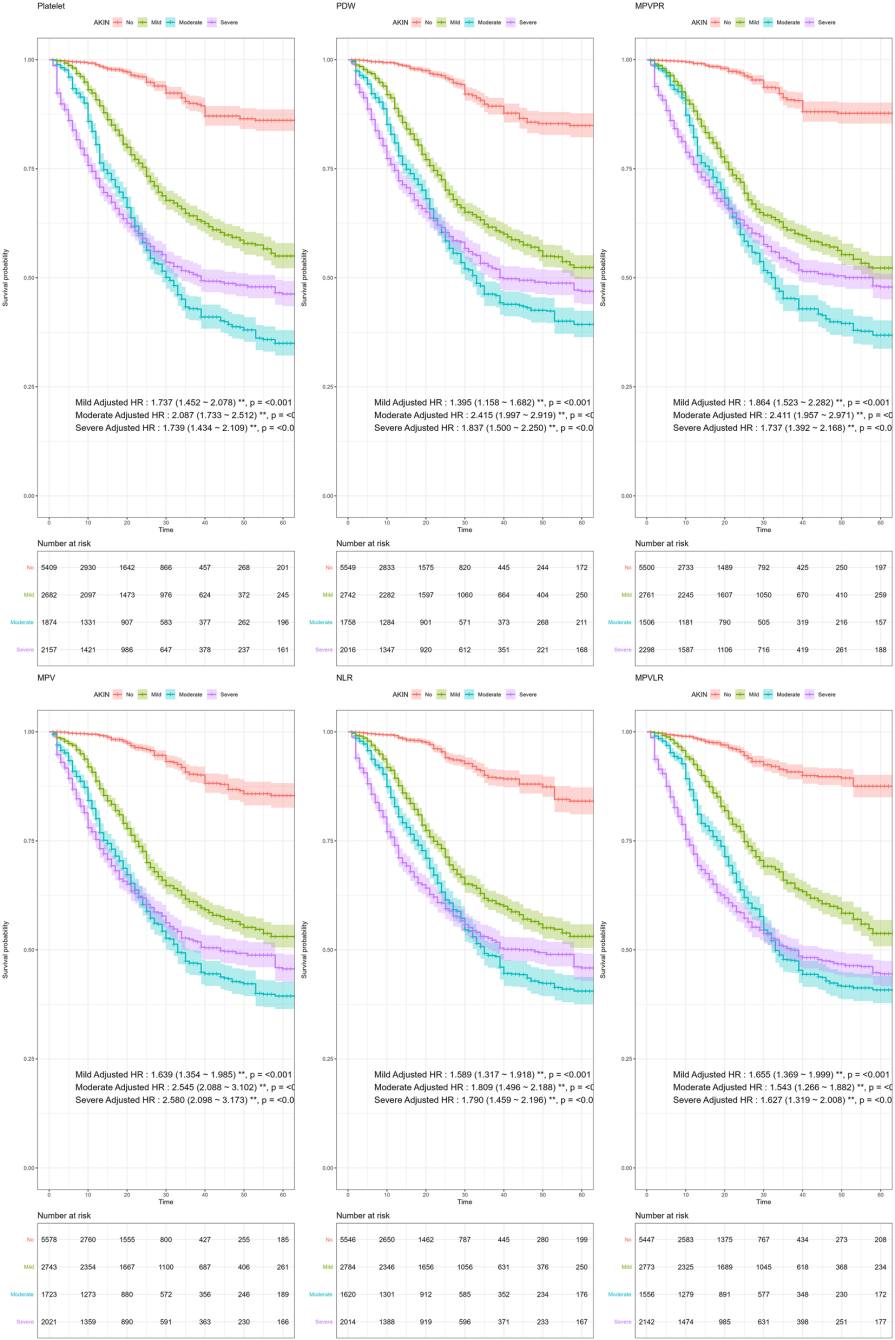
Adjusted Hazard Ratios (HR) for ARDS grades in predicting outcomes.

In the study, the severity of ARDS grades was determined using ordinal logistic regression modeling, with the adjusted Odds Ratios (ORs) detailed in Table S2. The mortality rate for each ARDS grade was predicted using the Cox proportional hazard model in conjunction with VGAM approach. Key findings included increased mortality risks associated with several markers: Platelet counts showed Hazard Ratios (HRs) of 1.737 in mild, 2.654 in moderate, and 1.739 in severe ARDS; MPV exhibited HRs of 1.639 in mild, 2.545 in moderate, and 2.580 in severe ARDS; and PDW had HRs of 1.395 in mild, 2.415 in moderate, and 1.837 in severe cases. Among ratio markers, NLR showed increased risk of mortality with HRs of 1.589 in mild, 1.809 in moderate, and 1.790 in severe ARDS; MPVPR presented HRs of 1.864 in mild, 2.411 in moderate, and 1.737 in severe; and MPVLR was associated with HRs of 1.655 in mild, 1.543 in moderate, and 1.627 in severe ARDS. These findings are detailed in Fig. 3 and Table S3, and the time-dependent changes of all CBC parameters according to ARDS grade are shown in Fig. S1.

## Discussion

Acute Respiratory Distress Syndrome (ARDS), a severe form of Acute Lung Injury, significantly impacts critically ill patients, carrying a high mortality rate^15^. Our study extensively investigates various hematological markers to enhance understanding of their role in assessing ARDS severity. We offer a comprehensive analysis of markers like NLR, MPVLR, Platelet count, PDW, and MPV for diagnosing and assessing ARDS severity. Our findings add to the growing evidence supporting the clinical utility of these markers, significantly impacting ARDS diagnosis and management.

Previous research has examined ARDS from different perspectives, including pathophysiology, ventilatory management, and pharmacological interventions^16,17^. Moreover, markers such as NLR and MPV, previously linked to conditions like cardiovascular disease and cancer^18,19^, and associated with inflammatory conditions^18,19^, have shown potential in enhancing existing diagnostic indicators, with MPV and PDW values at hospital admission effective in predicting mortality^20^. Our study extends this knowledge by applying these markers to ARDS diagnosis in burn patients, a context not extensively explored before.

We focus on specific ratio markers like NLR, MLR, PLR, and MPVPR, which have demonstrated utility in various medical conditions, and explore their innovative application to burn-induced ARDS. Specifically, the CBC data used for our analyses were those collected at the exact time of ARDS severity diagnosis, providing a critical insight into the condition at its clinically significant juncture. This approach presents a novel strategy in ARDS management, utilizing routine, cost-effective hematological markers to refine diagnostic and prognostic processes. By concentrating exclusively on burn patients, this research addresses a critical gap in medical practices, enhancing the early detection and severity assessment of ARDS.

These insights could significantly alter ARDS management in clinical settings. While our findings suggest that CBC tests could complement existing diagnostic tools by providing additional data points, it is essential to conduct further studies to evaluate whether these markers can truly simplify the diagnostic process and enable earlier interventions compared to traditional methods like the P/F ratio. Such advancements are crucial for improving outcomes in ARDS management, particularly in burn patients, and could be readily implemented in similar clinical environments.

Furthermore, this study contributes to the evolving field of personalized medicine in critical care. By exploring the relationship between specific hematological markers and ARDS severity in burn-induced cases, our research supports the development of customized treatment strategies tailored to individual patient responses. These findings suggest promising approaches for alleviating the significant healthcare burdens associated with ARDS, including high morbidity and mortality rates, extensive hospital stays, and substantial healthcare costs.

However, while our research indicates potential for more efficient resource management—potentially leading to shorter hospital stays and reduced intensive care requirements—these outcomes have not been empirically demonstrated within the scope of this study. It is essential that future research empirically investigates these potential benefits to fully understand the practical applications of hematological markers in ARDS management.

It is also crucial to note that our findings are derived from a cohort limited to burn-induced ARDS. Therefore, caution should be exercised when considering the application of these results to ARDS arising from other causes, as different etiologies may affect the efficacy of these markers. To enhance the generalizability of our results, further studies are necessary to validate these markers across a broader spectrum of ARDS etiologies. This would help substantiate the utility of these markers and support their broader application in the ICU, ensuring that our insights can effectively inform clinical practice across diverse ARDS populations.

The study's strengths include its methodological rigor and innovative approach. Meticulous data management, adherence to good clinical practices, and advanced statistical analyses, such as ordinal logistic regression modeling and Cox proportional hazard analysis, significantly enhance the credibility of its findings.

However, the study also presents certain limitations. Primarily, its retrospective design and the focus on a single hospital's patient population might limit the generalizability of the results. This research was conducted in a specialized burn unit, which may not fully represent broader ARDS populations or those resulting from different etiologies such as sepsis or trauma. These factors suggest that while our findings are robust for our study environment, they may not be directly applicable to other settings without further validation.

Moreover, the exclusive focus on burn-induced ARDS, while beneficial for in-depth analysis, restricts our ability to generalize these results to all ARDS cases. Future research should therefore aim to include a more diverse patient base across multiple centers and might consider employing a prospective design to confirm these findings and possibly reveal new insights.

## Conclusions

Our study indicates that hematological markers such as NLR, MPVLR, Platelet count, PDW, and MPV are valuable in diagnosing and determining the severity of ARDS. These findings are important for both current medical practices and future research, with the potential to improve patient outcomes and optimize resource utilization in critical care settings. However, the extent to which these markers can transform ARDS management needs further exploration. Incorporating these markers into clinical practice may enhance the precision of diagnoses and allow for more tailored treatment strategies, but additional research is necessary to fully establish their effectiveness and practical benefits in diverse clinical scenarios.

### Supplementary Information

Supplementary Information.

## Data Availability

The datasets used and/or analyzed during the current study are available from the corresponding author upon reasonable request.
