# Supplementary material for: A deep dive into burn-mediated ARDS severity assessment: a retrospective study on hematological markers
Source: Sci Rep. 2024 Jun 5;14:12873. doi: 10.1038/s41598-024-62235-4 (PMC11150426; doi:10.1038/s41598-024-62235-4)

Supplementary material

A Deep Dive into Burn-Mediated ARDS Severity Assessment: A Retrospective Study on Hematological Markers

Jeongsoo Park1, Dohern Kym,1,2*, Jun Hur1,2*, Jaechul Yoon1, Myongjin Kim1, Yong Suk Cho1,2, Wook Chun1,2, Dogeon Yoon2

1 Department of Surgery and Critical Care, Burn Center, Hangang Sacred Heart Hospital, Hallym University Medical Center, 12, Beodeunaru-ro 7-gil, Youngdeungpo-gu, Seoul, Korea, 07247

2 Burn Institutes, Hangang Sacred Heart Hospital, Hallym University Medical Center, 12, Beodeunaru-ro 7-gil, Youngdeungpo-gu, Seoul, Korea, 07247

*These authors contributed equally as Corresponding authors.

Department of Surgery and Critical Care, Burn Center, Hangang Sacred Heart Hospital, College of Medicine, Hallym University 12, Beodeunaru-ro 7-gil, Youngdeungpo-gu, Seoul, Korea, 07247

Tel. 82-2-2639-5446, Fax. 82-2-2678-4386, E-mail: hammerj@hallym.or.kr, dohern@hallym.or.kr

Contents

STROBE Statement—Checklist of items that should be included in reports of cohort studies 3

Table S1. Performance of CBC parameters Predicting AKI: AUC Analysis 6

Table S2. Odds Ratios for AKIN Severity Predictions using VGAM 10

Table S3. Adjusted HR for Mortality using VGAM Model in Enrolled Patients 14

Figure S1. Repaeted Measures over Time 17

1) WBC 17

2) Neutrophil 18

3) Lymphocyte 19

4) Monocyte 20

5) Eosinophil 21

6) Basophil 22

7) Immature Granulocyte 23

8) RBC 24

9) RDW 25

10) Hct 26

11) Hb 27

12 MCV 28

13) MCH 29

14) MCHC 30

15) Platelet 31

16) MPV 32

17) PDW 33

18) PCT 34

19) NLR 35

20 PLR 36

21 MLR 37

22) SII 38

23) MPVPR 39

24) MPVLR 40

25) MPVMR 41

26) MPVNR 42

# STROBE Statement—Checklist of items that should be included in reports of cohort studies

|  | | Item No | | Recommendation | Checked | |  |
| --- | --- | --- | --- | --- | --- | --- | --- |
| **Title and abstract** | | 1 | | (*a*) Indicate the study’s design with a commonly used term in the title or the abstract | Yes | |  |
|  |  |  |  | (*b*) Provide in the abstract an informative and balanced summary of what was done and what was found | Yes | |  |
| Introduction | | | | | | |  |
| Background/rationale | | 2 | | Explain the scientific background and rationale for the investigation being reported | Yes | |  |
| Objectives | | 3 | | State specific objectives, including any prespecified hypotheses | Yes | |  |
| Methods | | | | | | |  |
| Study design | | 4 | | Present key elements of study design early in the paper | Yes | |  |
| Setting | | 5 | | Describe the setting, locations, and relevant dates, including periods of recruitment, exposure, follow-up, and data collection | Yes | |  |
| Participants | | 6 | | (*a*) Give the eligibility criteria, and the sources and methods of selection of participants. Describe methods of follow-up | Yes | |  |
|  |  |  |  | (*b*) For matched studies, give matching criteria and number of exposed and unexposed | Yes | |  |
| Variables | | 7 | | Clearly define all outcomes, exposures, predictors, potential confounders, and effect modifiers. Give diagnostic criteria, if applicable | Yes | |  |
| Data sources/ measurement | | 8* | | For each variable of interest, give sources of data and details of methods of assessment (measurement). Describe comparability of assessment methods if there is more than one group | Yes | |  |
| Bias | | 9 | | Describe any efforts to address potential sources of bias | Yes | |  |
| Study size | | 10 | | Explain how the study size was arrived at | No | |  |
| Quantitative variables | | 11 | | Explain how quantitative variables were handled in the analyses. If applicable, describe which groupings were chosen and why | Yes | |  |
| Statistical methods | | 12 | | (*a*) Describe all statistical methods, including those used to control for confounding | Yes | |  |
|  |  |  |  | (*b*) Describe any methods used to examine subgroups and interactions | Yes | |  |
|  |  |  |  | (*c*) Explain how missing data were addressed | Yes | |  |
|  |  |  |  | (*d*) If applicable, explain how loss to follow-up was addressed | NA | |  |
|  |  |  |  | (*e*) Describe any sensitivity analyses | Yes | |  |
| Results | | | | |  | |  |
| Participants | | 13* | | (a) Report numbers of individuals at each stage of study—eg numbers potentially eligible, examined for eligibility, confirmed eligible, included in the study, completing follow-up, and analysed | Yes | |  |
|  |  |  |  | (b) Give reasons for non-participation at each stage | NA | |  |
|  |  |  |  | (c) Consider use of a flow diagram | Yes | |  |
| Descriptive data | | 14* | | (a) Give characteristics of study participants (eg demographic, clinical, social) and information on exposures and potential confounders | Yes | |  |
|  |  |  |  | (b) Indicate number of participants with missing data for each variable of interest | Yes | |  |
|  |  |  |  | (c) Summarise follow-up time (eg, average and total amount) | NA | |  |
| Outcome data | | 15* | | Report numbers of outcome events or summary measures over time | Yes | |  |
| Main results | 16 | | (*a*) Give unadjusted estimates and, if applicable, confounder-adjusted estimates and their precision (eg, 95% confidence interval). Make clear which confounders were adjusted for and why they were included | | | Yes | |
|  |  |  | (*b*) Report category boundaries when continuous variables were categorized | | | Yes | |
|  |  |  | (*c*) If relevant, consider translating estimates of relative risk into absolute risk for a meaningful time period | | | Yes | |
| Other analyses | 17 | | Report other analyses done—eg analyses of subgroups and interactions, and sensitivity analyses | | | Yes | |
| Discussion | | | | | | | |
| Key results | 18 | | Summarise key results with reference to study objectives | | | Yes | |
| Limitations | 19 | | Discuss limitations of the study, taking into account sources of potential bias or imprecision. Discuss both direction and magnitude of any potential bias | | | Yes | |
| Interpretation | 20 | | Give a cautious overall interpretation of results considering objectives, limitations, multiplicity of analyses, results from similar studies, and other relevant evidence | | | Yes | |
| Generalisability | 21 | | Discuss the generalisability (external validity) of the study results | | | Yes | |
| Other information | | | | | | | |
| Funding | 22 | | Give the source of funding and the role of the funders for the present study and, if applicable, for the original study on which the present article is based | | | Yes | |

*Give information separately for exposed and unexposed groups.

**Note:** An Explanation and Elaboration article discusses each checklist item and gives methodological background and published examples of transparent reporting. The STROBE checklist is best used in conjunction with this article (freely available on the Web sites of PLoS Medicine at http://www.plosmedicine.org/, Annals of Internal Medicine at http://www.annals.org/, and Epidemiology at http://www.epidem.com/). Information on the STROBE Initiative is available at http://www.strobe-statement.org.

# Table S1. Performance of CBC parameters Diagnosing ARDS: AUC Analysis

| CBC parameters | Variables | AUC (95% CI) | Optimal cut-off | Accurarcy (95% CI) | Sensitivity (95% CI) | Specificity (95% CI) | PPV (95% CI) | NPV (95% CI) |
| --- | --- | --- | --- | --- | --- | --- | --- | --- |
| WBC-realated | WBC | 0.607 (0.584 ~ 0.629) | 12.64 | 0.613 (0.595 ~ 0.631) | 0.518 (0.488 ~ 0.549) | 0.673 (0.650 ~ 0.696) | 0.502 (0.472 ~ 0.532) | 0.687 (0.664 ~ 0.710) |
|  | Neutrophil | 0.656 (0.634 ~ 0.677) | 8.31 | 0.646 (0.628 ~ 0.664) | 0.557 (0.527 ~ 0.587) | 0.702 (0.679 ~ 0.724) | 0.543 (0.512 ~ 0.572) | 0.714 (0.692 ~ 0.736) |
|  | Lymphocyte | 0.671 (0.649 ~ 0.692) | 0.98 | 0.598 (0.579 ~ 0.616) | 0.711 (0.683 ~ 0.739) | 0.526 (0.502 ~ 0.550) | 0.488 (0.463 ~ 0.513) | 0.742 (0.716 ~ 0.766) |
|  | Monocyte | 0.544 (0.521 ~ 0.567) | 0.40 | 0.505 (0.486 ~ 0.523) | 0.599 (0.569 ~ 0.628) | 0.445 (0.421 ~ 0.469) | 0.406 (0.382 ~ 0.431) | 0.636 (0.608 ~ 0.664) |
|  | Eosinophil | 0.620 (0.599 ~ 0.642) | 0.17 | 0.570 (0.552 ~ 0.589) | 0.705 (0.676 ~ 0.732) | 0.487 (0.462 ~ 0.511) | 0.461 (0.437 ~ 0.486) | 0.725 (0.698 ~ 0.751) |
|  | Basophil | 0.558 (0.535 ~ 0.581) | 0.05 | 0.580 (0.560 ~ 0.599) | 0.464 (0.433 ~ 0.496) | 0.657 (0.632 ~ 0.681) | 0.473 (0.441 ~ 0.505) | 0.649 (0.624 ~ 0.673) |
|  | Immature Granulocyte | 0.717 (0.687 ~ 0.747) | 0.07 | 0.664 (0.635 ~ 0.691) | 0.397 (0.349 ~ 0.445) | 0.823 (0.793 ~ 0.851) | 0.573 (0.514 ~ 0.631) | 0.695 (0.663 ~ 0.727) |
| RBC-realated | RBC | 0.555 (0.532 ~ 0.577) | 3.78 | 0.538 (0.519 ~ 0.556) | 0.625 (0.595 ~ 0.654) | 0.482 (0.458 ~ 0.506) | 0.434 (0.409 ~ 0.459) | 0.669 (0.642 ~ 0.696) |
|  | RDW | 0.706 (0.687 ~ 0.726) | 13.70 | 0.668 (0.650 ~ 0.686) | 0.650 (0.620 ~ 0.678) | 0.680 (0.657 ~ 0.702) | 0.563 (0.535 ~ 0.591) | 0.753 (0.731 ~ 0.775) |
|  | Hct | 0.525 (0.503 ~ 0.547) | 34.10 | 0.521 (0.502 ~ 0.540) | 0.595 (0.565 ~ 0.624) | 0.474 (0.450 ~ 0.498) | 0.418 (0.393 ~ 0.443) | 0.648 (0.621 ~ 0.675) |
|  | Hb | 0.554 (0.532 ~ 0.577) | 10.70 | 0.538 (0.519 ~ 0.556) | 0.636 (0.606 ~ 0.665) | 0.475 (0.451 ~ 0.499) | 0.435 (0.410 ~ 0.460) | 0.673 (0.645 ~ 0.699) |
|  | MCV | 0.584 (0.562 ~ 0.605) | 92.30 | 0.566 (0.547 ~ 0.584) | 0.555 (0.524 ~ 0.585) | 0.573 (0.549 ~ 0.597) | 0.452 (0.425 ~ 0.479) | 0.669 (0.644 ~ 0.694) |
|  | MCH | 0.547 (0.525 ~ 0.569) | 29.80 | 0.519 (0.500 ~ 0.538) | 0.527 (0.496 ~ 0.557) | 0.514 (0.490 ~ 0.538) | 0.408 (0.382 ~ 0.434) | 0.631 (0.605 ~ 0.657) |
|  | MCHC | 0.510 (0.487 ~ 0.532) | 33.70 | 0.513 (0.494 ~ 0.532) | 0.519 (0.489 ~ 0.549) | 0.509 (0.485 ~ 0.534) | 0.402 (0.376 ~ 0.428) | 0.625 (0.599 ~ 0.651) |
| Platelet-realated | Platelet | 0.755 (0.736 ~ 0.774) | 174.00 | 0.637 (0.618 ~ 0.655) | 0.783 (0.757 ~ 0.808) | 0.543 (0.519 ~ 0.567) | 0.521 (0.497 ~ 0.546) | 0.798 (0.773 ~ 0.821) |
|  | MPV | 0.739 (0.721 ~ 0.758) | 10.14 | 0.687 (0.669 ~ 0.704) | 0.686 (0.657 ~ 0.713) | 0.687 (0.664 ~ 0.709) | 0.582 (0.554 ~ 0.609) | 0.775 (0.753 ~ 0.796) |
|  | PDW | 0.753 (0.734 ~ 0.772) | 10.90 | 0.707 (0.689 ~ 0.724) | 0.644 (0.614 ~ 0.672) | 0.747 (0.725 ~ 0.767) | 0.617 (0.588 ~ 0.646) | 0.768 (0.746 ~ 0.788) |
|  | PCT | 0.714 (0.694 ~ 0.733) | 0.15 | 0.594 (0.576 ~ 0.613) | 0.819 (0.794 ~ 0.842) | 0.452 (0.428 ~ 0.476) | 0.486 (0.463 ~ 0.509) | 0.798 (0.771 ~ 0.823) |
| Ratios | NLR | 0.816 (0.800 ~ 0.833) | 8.06 | 0.771 (0.754 ~ 0.786) | 0.649 (0.620 ~ 0.678) | 0.847 (0.829 ~ 0.864) | 0.730 (0.700 ~ 0.758) | 0.792 (0.773 ~ 0.811) |
|  | PLR | 0.500 (0.478 ~ 0.523) | 462.50 | 0.533 (0.514 ~ 0.551) | 0.411 (0.381 ~ 0.441) | 0.610 (0.586 ~ 0.633) | 0.401 (0.371 ~ 0.430) | 0.620 (0.596 ~ 0.643) |
|  | MLR | 0.688 (0.667 ~ 0.709) | 0.65 | 0.656 (0.638 ~ 0.674) | 0.565 (0.534 ~ 0.595) | 0.714 (0.692 ~ 0.736) | 0.556 (0.526 ~ 0.586) | 0.721 (0.699 ~ 0.743) |
|  | SII | 0.569 (0.546 ~ 0.591) | 2,200.09 | 0.589 (0.571 ~ 0.608) | 0.445 (0.414 ~ 0.475) | 0.681 (0.658 ~ 0.703) | 0.469 (0.438 ~ 0.501) | 0.659 (0.636 ~ 0.681) |
|  | MPVPR | 0.798 (0.781 ~ 0.815) | 0.07 | 0.760 (0.744 ~ 0.776) | 0.590 (0.560 ~ 0.620) | 0.868 (0.851 ~ 0.883) | 0.739 (0.708 ~ 0.768) | 0.769 (0.750 ~ 0.788) |
|  | MPVLR | 0.814 (0.797 ~ 0.831) | 11.20 | 0.768 (0.752 ~ 0.784) | 0.653 (0.623 ~ 0.681) | 0.842 (0.824 ~ 0.859) | 0.723 (0.694 ~ 0.752) | 0.793 (0.773 ~ 0.811) |
|  | MPVMR | 0.697 (0.676 ~ 0.719) | 22.37 | 0.695 (0.678 ~ 0.713) | 0.543 (0.513 ~ 0.574) | 0.792 (0.772 ~ 0.811) | 0.623 (0.591 ~ 0.654) | 0.732 (0.711 ~ 0.753) |
|  | MPVNR | 0.514 (0.491 ~ 0.537) | 2.37 | 0.546 (0.527 ~ 0.565) | 0.394 (0.364 ~ 0.424) | 0.643 (0.619 ~ 0.666) | 0.411 (0.381 ~ 0.442) | 0.626 (0.602 ~ 0.649) |
| The darkest color represents an AUC > 0.80. ; The middle color represents an AUC > 0.75. ; The lightest color represents an AUC > 0.70 | | | | | | | | |

# Table S2. Odds Ratios for ARDS Severity Predictions using VGAM

| CBC parameters | Variables | AKIN 1   adjusted OR (95%CI) | AKIN 1   adjusted OR (95%CI) over time | AKIN 2   adjusted OR (95%CI) | AKIN 2   adjusted OR (95%CI) over time | AKIN 3   adjusted OR (95%CI) | AKIN 3   adjusted OR (95%CI) over time | RMSE(Rank) |
| --- | --- | --- | --- | --- | --- | --- | --- | --- |
| WBC-realated | WBC | 1.140 (1.113 - 1.169) ** | 0.948 (0.926 - 0.971) ** | 1.138 (1.110 - 1.168) ** | 1.006 (0.981 - 1.032) | 1.165 (1.124 - 1.207) ** | 1.030 (0.994 - 1.067) | 1.79(8) |
|  | Neutrophil | 1.201 (1.172 - 1.231) ** | 0.979 (0.956 - 1.003) | 1.175 (1.146 - 1.206) ** | 1.030 (1.004 - 1.056) * | 1.189 (1.147 - 1.232) ** | 1.045 (1.008 - 1.083) * | 1.82(13) |
|  | Lymphocyte | 0.852 (0.852 - 0.852) ** | 0.920 (0.920 - 0.920) ** | 0.942 (0.942 - 0.942) ** | 0.972 (0.972 - 0.972) ** | 1.045 (1.016 - 1.074) * | 1.073 (1.043 - 1.104) ** | 1.59(2) |
|  | Monocyte | 1.078 (1.052 - 1.104) ** | 0.925 (0.903 - 0.947) ** | 1.078 (1.051 - 1.105) ** | 0.965 (0.940 - 0.990) * | 1.081 (1.043 - 1.120) ** | 0.999 (0.963 - 1.037) | 1.8(11) |
|  | Eosinophil | 0.837 (0.815 - 0.860) ** | 0.997 (0.972 - 1.024) | 0.879 (0.853 - 0.905) ** | 0.998 (0.969 - 1.027) | 0.929 (0.889 - 0.970) ** | 1.001 (0.958 - 1.046) | 1.85(19) |
|  | Basophil | 0.995 (0.969 - 1.023) | 0.963 (0.938 - 0.988) * | 1.023 (0.994 - 1.052) | 0.994 (0.967 - 1.022) | 1.036 (0.995 - 1.078) | 1.035 (0.995 - 1.076) | 1.76(6) |
|  | Immature Granulocyte | 1.191 (1.146 - 1.237) ** | 0.999 (0.963 - 1.036) | 1.121 (1.078 - 1.166) ** | 1.011 (0.973 - 1.050) | 1.124 (1.067 - 1.185) ** | 1.050 (0.997 - 1.106) | 1.76(5) |
| RBC-realated | RBC | 1.041 (1.016 - 1.067) * | 0.887 (0.866 - 0.908) ** | 1.068 (1.041 - 1.095) ** | 0.948 (0.925 - 0.972) ** | 1.062 (1.025 - 1.101) ** | 1.006 (0.971 - 1.043) | 1.75(4) |
|  | RDW | 1.225 (1.194 - 1.257) ** | 0.988 (0.964 - 1.012) | 1.123 (1.092 - 1.154) ** | 1.006 (0.980 - 1.032) | 1.129 (1.086 - 1.174) ** | 1.036 (0.998 - 1.075) | 1.83(17) |
|  | Hct | 1.076 (1.050 - 1.102) ** | 0.886 (0.865 - 0.908) ** | 1.100 (1.072 - 1.129) ** | 0.944 (0.921 - 0.969) ** | 1.088 (1.049 - 1.128) ** | 1.009 (0.972 - 1.047) | 1.81(12) |
|  | Hb | 1.056 (1.030 - 1.082) ** | 0.885 (0.864 - 0.906) ** | 1.075 (1.048 - 1.103) ** | 0.946 (0.922 - 0.970) ** | 1.058 (1.020 - 1.097) * | 1.001 (0.965 - 1.038) | 1.82(14) |
|  | MCV | 1.132 (1.104 - 1.160) ** | 0.975 (0.952 - 0.999) * | 1.120 (1.091 - 1.150) ** | 1.013 (0.987 - 1.040) | 1.086 (1.045 - 1.128) ** | 1.056 (1.016 - 1.097) * | 1.83(18) |
|  | MCH | 1.109 (1.082 - 1.136) ** | 1.025 (1.001 - 1.050) * | 1.101 (1.072 - 1.130) ** | 1.043 (1.016 - 1.071) * | 1.031 (0.992 - 1.070) | 1.063 (1.023 - 1.104) * | 1.82(16) |
|  | MCHC | 1.040 (1.015 - 1.065) * | 1.066 (1.041 - 1.092) ** | 1.030 (1.004 - 1.057) * | 1.048 (1.022 - 1.075) ** | 1.002 (0.966 - 1.039) | 1.020 (0.984 - 1.058) | 1.8(10) |
| Platelet-realated | Platelet | 0.723 (0.706 - 0.741) ** | 0.968 (0.946 - 0.991) * | 0.808 (0.790 - 0.826) ** | 0.974 (0.951 - 0.998) * | 0.913 (0.883 - 0.944) ** | 0.980 (0.947 - 1.014) | 1.58(1) |
|  | MPV | 1.385 (1.351 - 1.421) ** | 1.034 (1.009 - 1.060) * | 1.243 (1.211 - 1.276) ** | 1.034 (1.007 - 1.061) * | 1.219 (1.175 - 1.266) ** | 0.997 (0.960 - 1.034) | 1.97(24) |
|  | PDW | 1.417 (1.382 - 1.453) ** | 1.034 (1.008 - 1.060) * | 1.269 (1.236 - 1.302) ** | 1.027 (1.001 - 1.053) * | 1.174 (1.132 - 1.218) ** | 1.006 (0.970 - 1.043) | 1.93(23) |
|  | PCT | 0.761 (0.745 - 0.777) ** | 0.966 (0.947 - 0.985) ** | 0.837 (0.821 - 0.854) ** | 0.971 (0.954 - 0.988) ** | 0.951 (0.921 - 0.982) * | 0.985 (0.955 - 1.017) | 1.6(3) |
| Ratios | NLR | 1.464 (1.426 - 1.503) ** | 1.081 (1.054 - 1.109) ** | 1.263 (1.231 - 1.295) ** | 1.053 (1.027 - 1.079) ** | 1.212 (1.171 - 1.254) ** | 1.012 (0.979 - 1.046) | 2.08(26) |
|  | PLR | 0.949 (0.926 - 0.972) ** | 1.072 (1.047 - 1.098) ** | 0.954 (0.930 - 0.979) ** | 1.057 (1.030 - 1.085) ** | 0.991 (0.955 - 1.028) | 1.017 (0.979 - 1.056) | 1.82(15) |
|  | MLR | 1.396 (1.362 - 1.431) ** | 1.035 (1.010 - 1.061) * | 1.234 (1.204 - 1.266) ** | 1.025 (0.999 - 1.051) | 1.154 (1.113 - 1.195) ** | 0.994 (0.959 - 1.031) | 1.87(21) |
|  | SII | 1.068 (1.043 - 1.094) ** | 1.036 (1.011 - 1.061) * | 1.063 (1.037 - 1.091) ** | 1.044 (1.018 - 1.071) ** | 1.094 (1.056 - 1.133) ** | 1.019 (0.983 - 1.056) | 1.8(9) |
|  | MPVPR | 1.492 (1.451 - 1.534) ** | 1.026 (0.999 - 1.055) | 1.268 (1.236 - 1.301) ** | 1.020 (0.995 - 1.045) | 1.175 (1.136 - 1.216) ** | 1.016 (0.984 - 1.050) | 1.9(22) |
|  | MPVLR | 1.483 (1.445 - 1.522) ** | 1.114 (1.086 - 1.142) ** | 1.243 (1.211 - 1.275) ** | 1.045 (1.020 - 1.071) ** | 1.164 (1.124 - 1.204) ** | 1.016 (0.982 - 1.050) | 2(25) |
|  | MPVMR | 1.192 (1.162 - 1.222) ** | 1.098 (1.071 - 1.125) ** | 1.122 (1.093 - 1.151) ** | 1.064 (1.037 - 1.091) ** | 1.099 (1.061 - 1.137) ** | 1.043 (1.008 - 1.080) * | 1.85(20) |
|  | MPVNR | 1.021 (0.997 - 1.046) | 1.036 (1.012 - 1.061) * | 1.012 (0.987 - 1.039) | 1.021 (0.995 - 1.048) | 0.959 (0.923 - 0.997) * | 1.047 (1.006 - 1.089) * | 1.78(7) |
| ** This is p-value < 0.001.; * This is p-value < 0.05 | | | | | | | | |
| The darkest color indicates the top 3 ranking based on the Root Mean Square Error (RMSE).; The middle color represents the top 4 to 6 rankings.; The lightest color represents the top 7 to 9 rankings | | | | | | | | |

# Table S3. Adjusted HR for Mortality using VGAM Model in Enrolled Patients

| CBC parameters | Variables | Mild   Adjusted Hazard Ratio (95%CI) | Moderate   Adjusted Hazard Ratio (95%CI) | Severe   Adjusted Hazard Ratio (95%CI) |
| --- | --- | --- | --- | --- |
| WBC-realated | WBC | 0.624 (0.527 ~ 0.740) ** | 0.912 (0.759 ~ 1.095) | 1.276 (1.068 ~ 1.526) * |
|  | Neutrophil | 0.746 (0.628 ~ 0.887) ** | 1.194 (0.991 ~ 1.438) | 1.508 (1.256 ~ 1.810) ** |
|  | Lymphocyte | 0.683 (0.575 ~ 0.813) ** | 0.709 (0.592 ~ 0.850) ** | 0.942 (0.781 ~ 1.137) |
|  | Monocyte | 0.562 (0.475 ~ 0.666) ** | 0.862 (0.719 ~ 1.033) | 0.930 (0.775 ~ 1.116) |
|  | Eosinophil | 0.662 (0.555 ~ 0.790) ** | 1.086 (0.905 ~ 1.304) | 0.976 (0.804 ~ 1.183) |
|  | Basophil | 0.562 (0.469 ~ 0.673) ** | 0.715 (0.587 ~ 0.870) ** | 0.876 (0.722 ~ 1.062) |
|  | Immature Granulocyte | 0.782 (0.605 ~ 1.012) | 0.878 (0.662 ~ 1.164) | 1.238 (0.929 ~ 1.650) |
| RBC-realated | RBC | 0.576 (0.489 ~ 0.679) ** | 0.822 (0.693 ~ 0.975) * | 1.012 (0.846 ~ 1.210) |
|  | RDW | 1.046 (0.862 ~ 1.269) | 0.991 (0.807 ~ 1.218) | 1.465 (1.197 ~ 1.792) ** |
|  | Hct | 0.582 (0.496 ~ 0.684) ** | 0.822 (0.696 ~ 0.972) * | 1.045 (0.877 ~ 1.244) |
|  | Hb | 0.560 (0.476 ~ 0.659) ** | 0.815 (0.689 ~ 0.965) * | 0.961 (0.804 ~ 1.149) |
|  | MCV | 0.739 (0.622 ~ 0.879) ** | 1.140 (0.950 ~ 1.368) | 1.278 (1.060 ~ 1.540) * |
|  | MCH | 0.672 (0.567 ~ 0.796) ** | 0.975 (0.820 ~ 1.160) | 0.941 (0.778 ~ 1.138) |
|  | MCHC | 0.511 (0.431 ~ 0.605) ** | 0.659 (0.551 ~ 0.787) ** | 0.734 (0.610 ~ 0.882) ** |
| Platelet-realated | Platelet | 1.737 (1.452 ~ 2.078) ** | 2.087 (1.733 ~ 2.512) ** | 1.739 (1.434 ~ 2.109) ** |
|  | MPV | 1.639 (1.354 ~ 1.985) ** | 2.545 (2.088 ~ 3.102) ** | 2.580 (2.098 ~ 3.173) ** |
|  | PDW | 1.395 (1.158 ~ 1.682) ** | 2.415 (1.997 ~ 2.919) ** | 1.837 (1.500 ~ 2.250) ** |
|  | PCT | 1.136 (0.955 ~ 1.353) | 1.467 (1.226 ~ 1.756) ** | 1.286 (1.067 ~ 1.550) * |
| Ratios | NLR | 1.589 (1.317 ~ 1.918) ** | 1.809 (1.496 ~ 2.188) ** | 1.790 (1.459 ~ 2.196) ** |
|  | PLR | 0.526 (0.445 ~ 0.623) ** | 0.768 (0.644 ~ 0.915) * | 0.748 (0.620 ~ 0.902) * |
|  | MLR | 1.028 (0.868 ~ 1.217) | 1.335 (1.123 ~ 1.586) * | 1.325 (1.106 ~ 1.588) * |
|  | SII | 0.542 (0.456 ~ 0.644) ** | 0.699 (0.582 ~ 0.840) ** | 0.792 (0.658 ~ 0.954) * |
|  | MPVPR | 1.864 (1.523 ~ 2.282) ** | 2.411 (1.957 ~ 2.971) ** | 1.737 (1.392 ~ 2.168) ** |
|  | MPVLR | 1.655 (1.369 ~ 1.999) ** | 1.543 (1.266 ~ 1.882) ** | 1.627 (1.319 ~ 2.008) ** |
|  | MPVMR | 0.782 (0.651 ~ 0.939) * | 1.177 (0.969 ~ 1.429) | 1.067 (0.872 ~ 1.306) |
|  | MPVNR | 0.565 (0.473 ~ 0.674) ** | 0.819 (0.679 ~ 0.986) * | 0.794 (0.653 ~ 0.965) * |
| ** This is p-value < 0.001.; * This is p-value < 0.05 | | | | |
| The darker color represents an HR > 1 with a p-value < 0.05 in all three sections.; The lighter color indicates an HR > 1 with a p-value < 0.05 in two sections | | | | |

# Figure S1. Repaeted Measures over Time

## 1) WBC


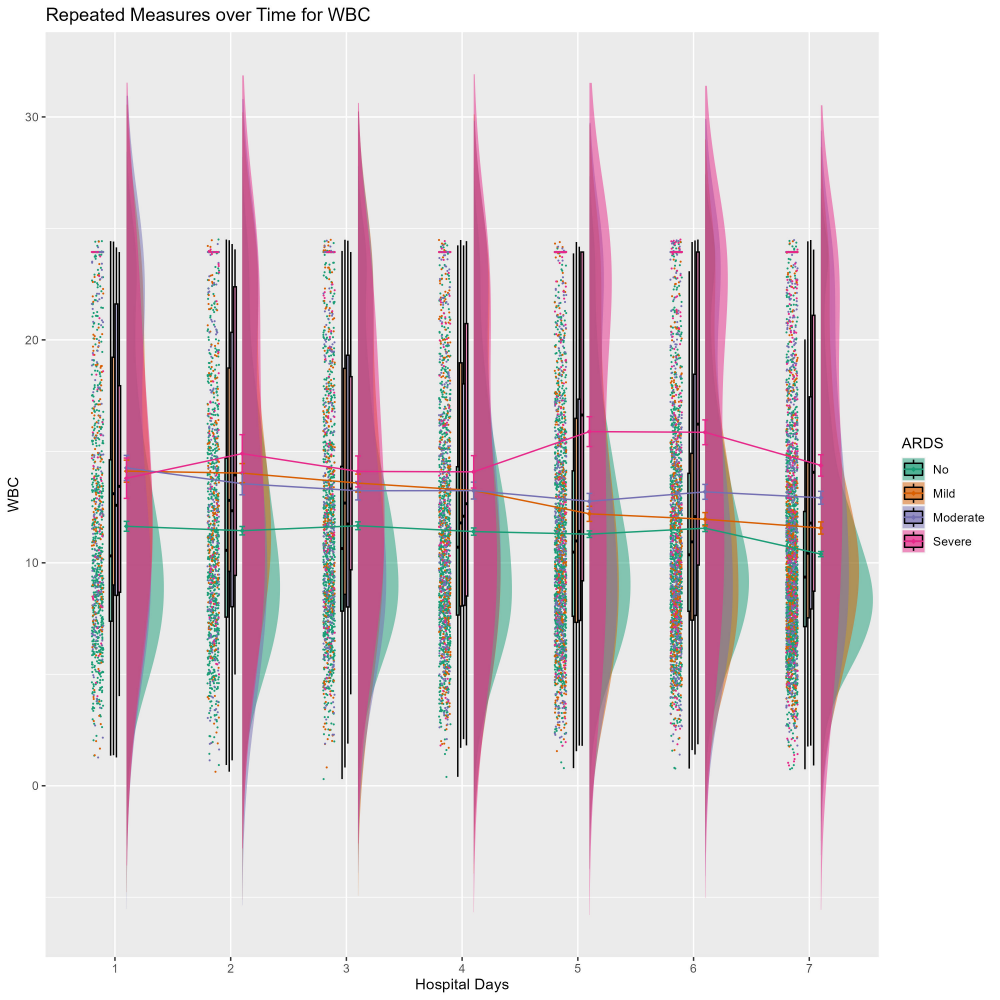


## 2) Neutrophil


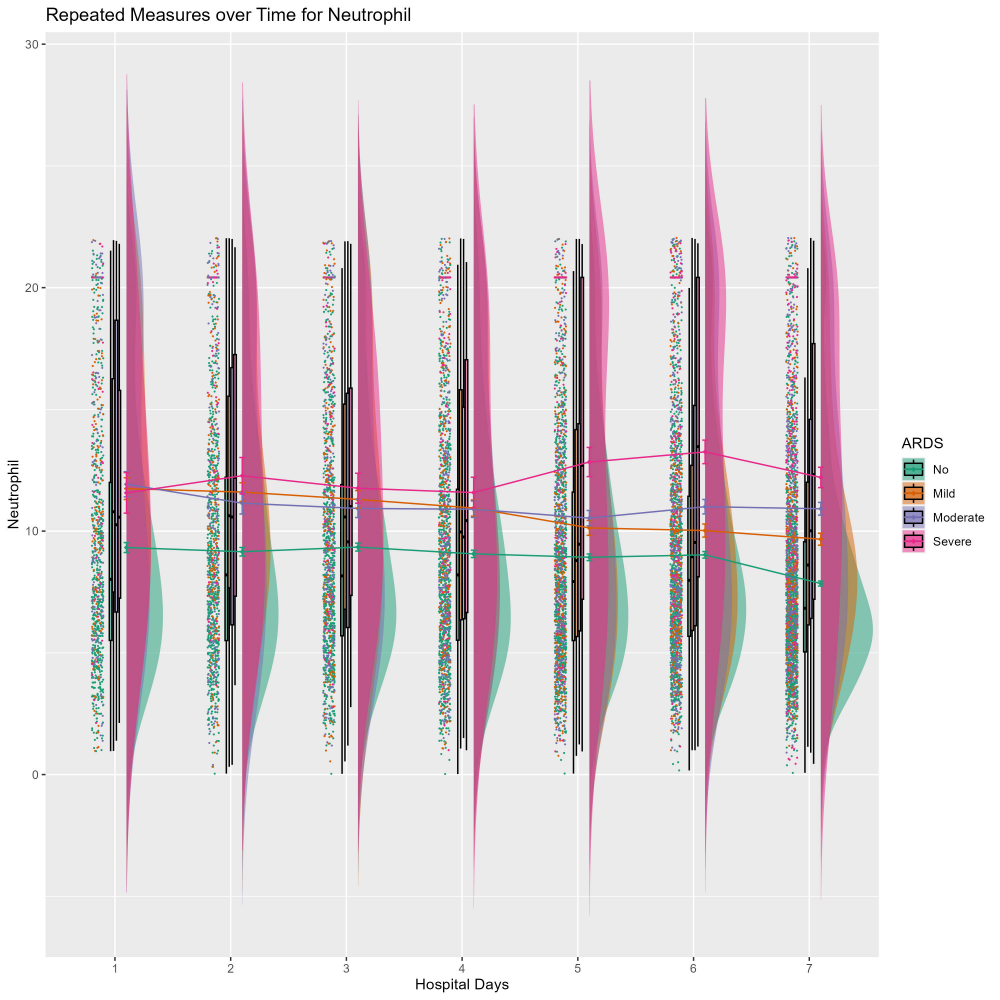


## 3) Lymphocyte


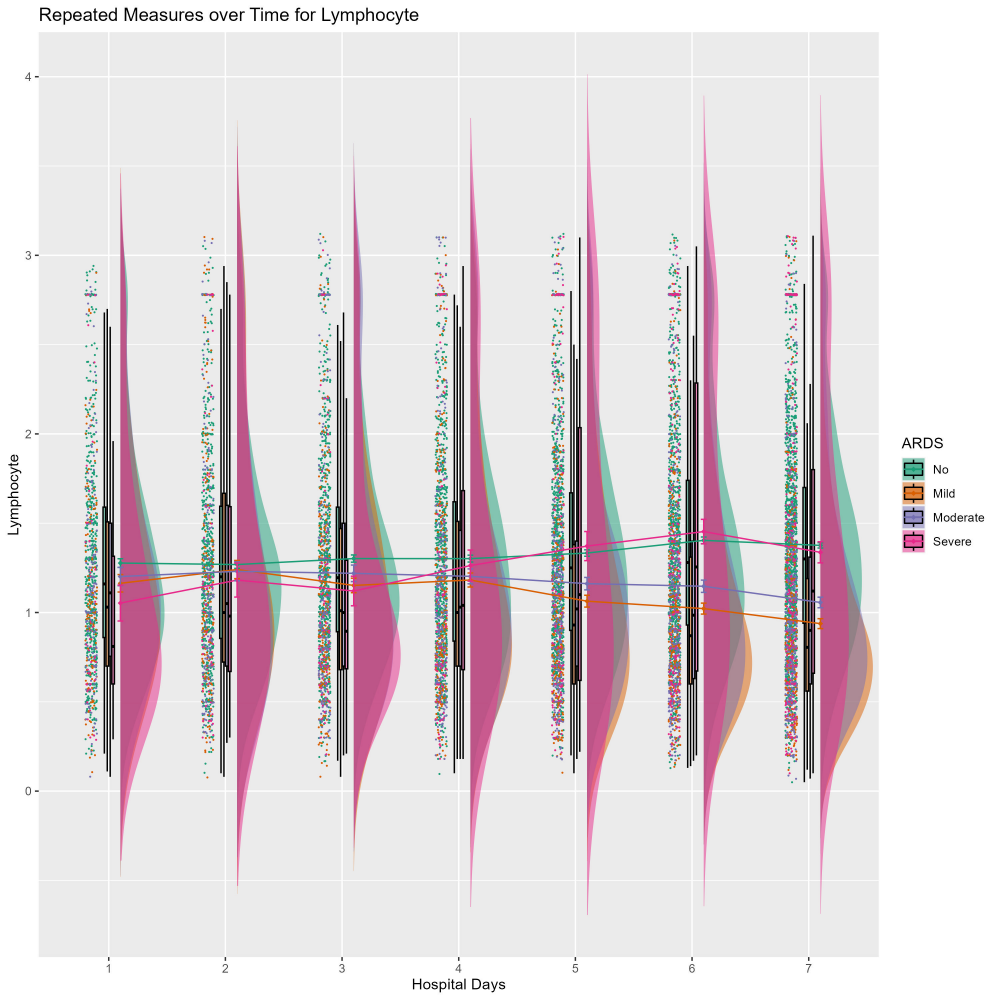


## 4) Monocyte


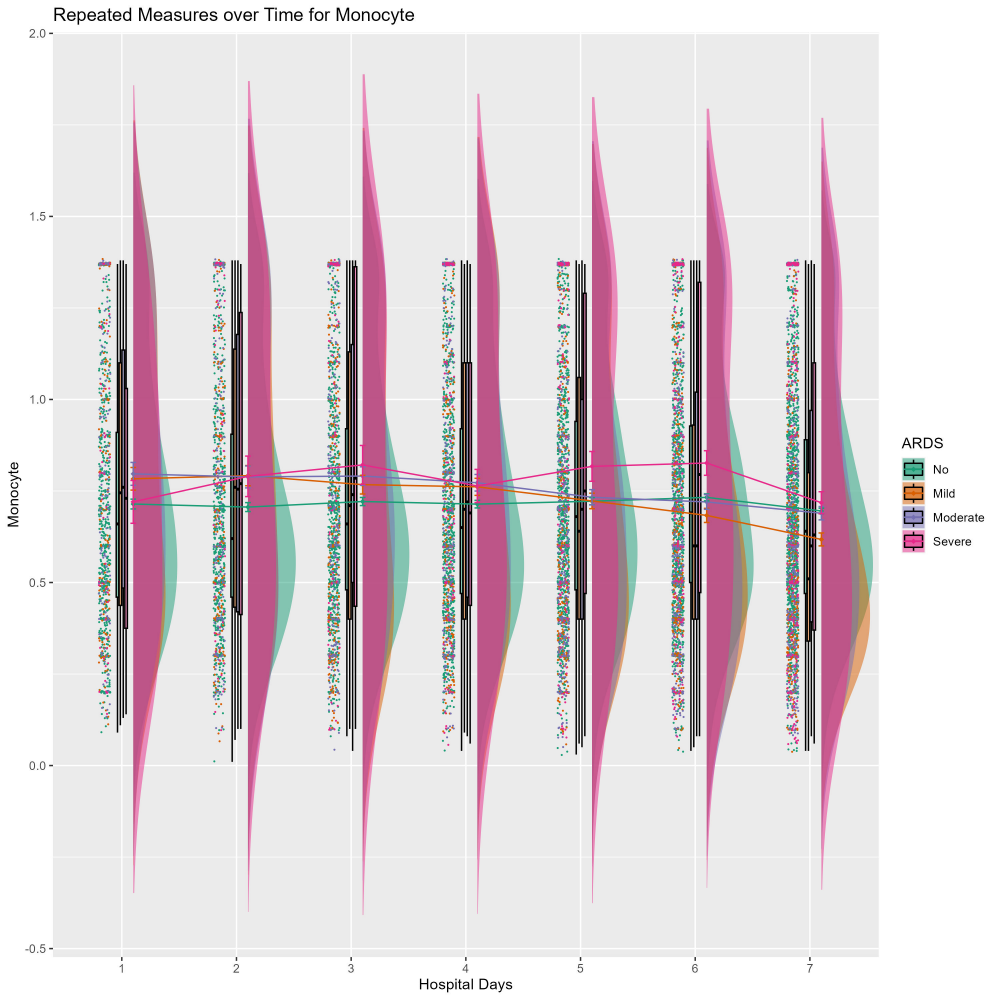


## 5) Eosinophil


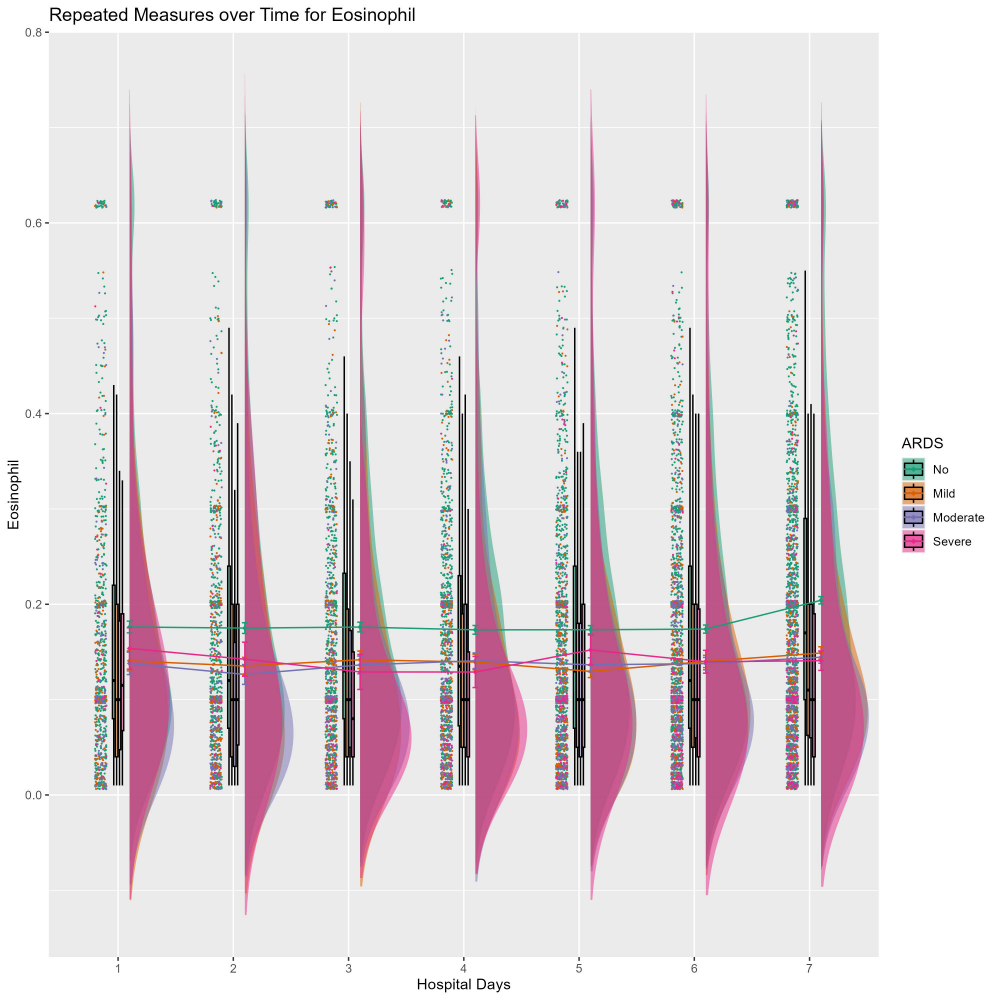


## 6) Basophil


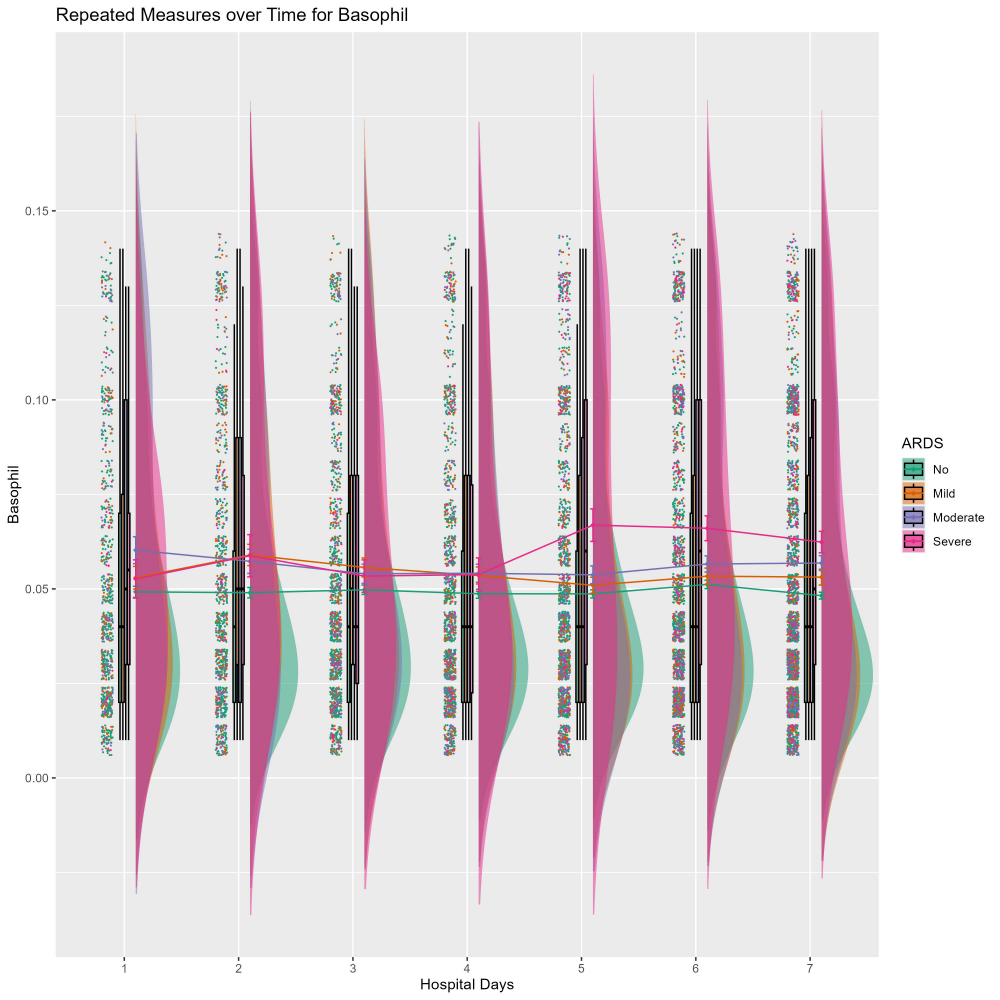


## 7) Immature Granulocyte


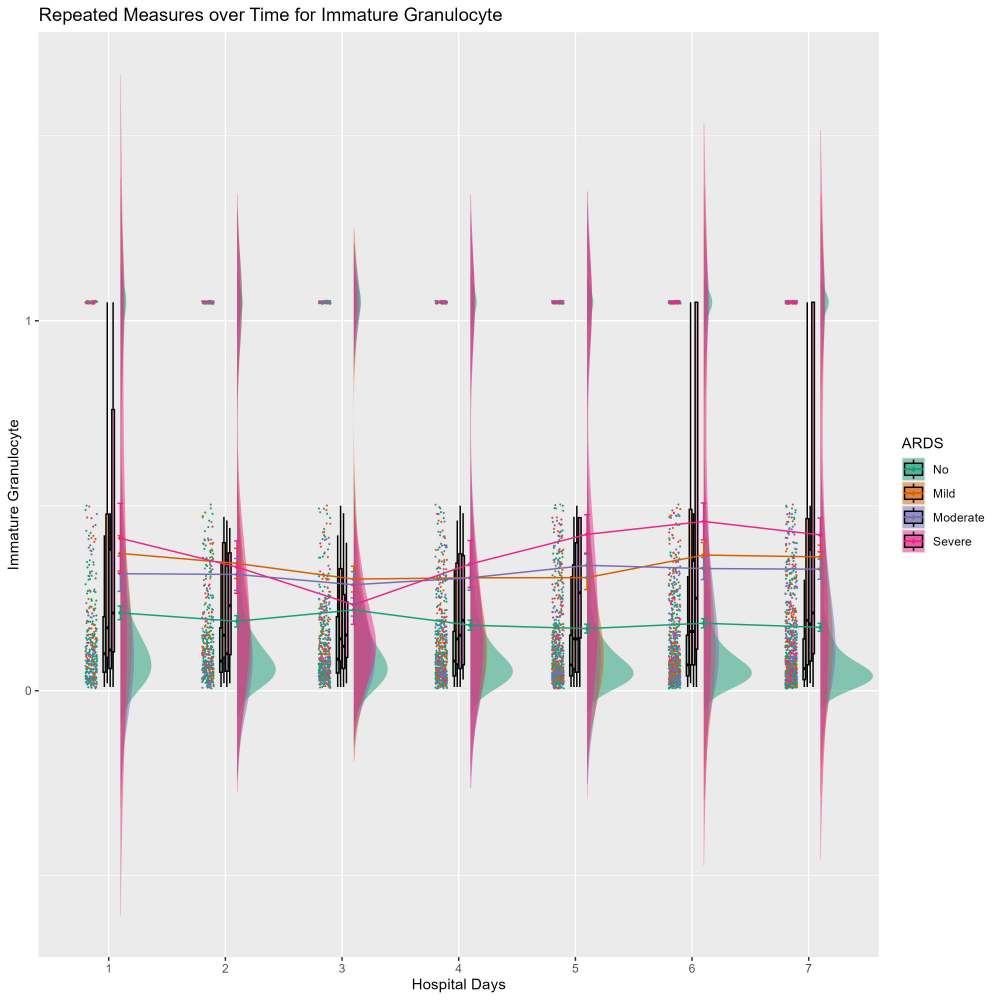


## 8) RBC


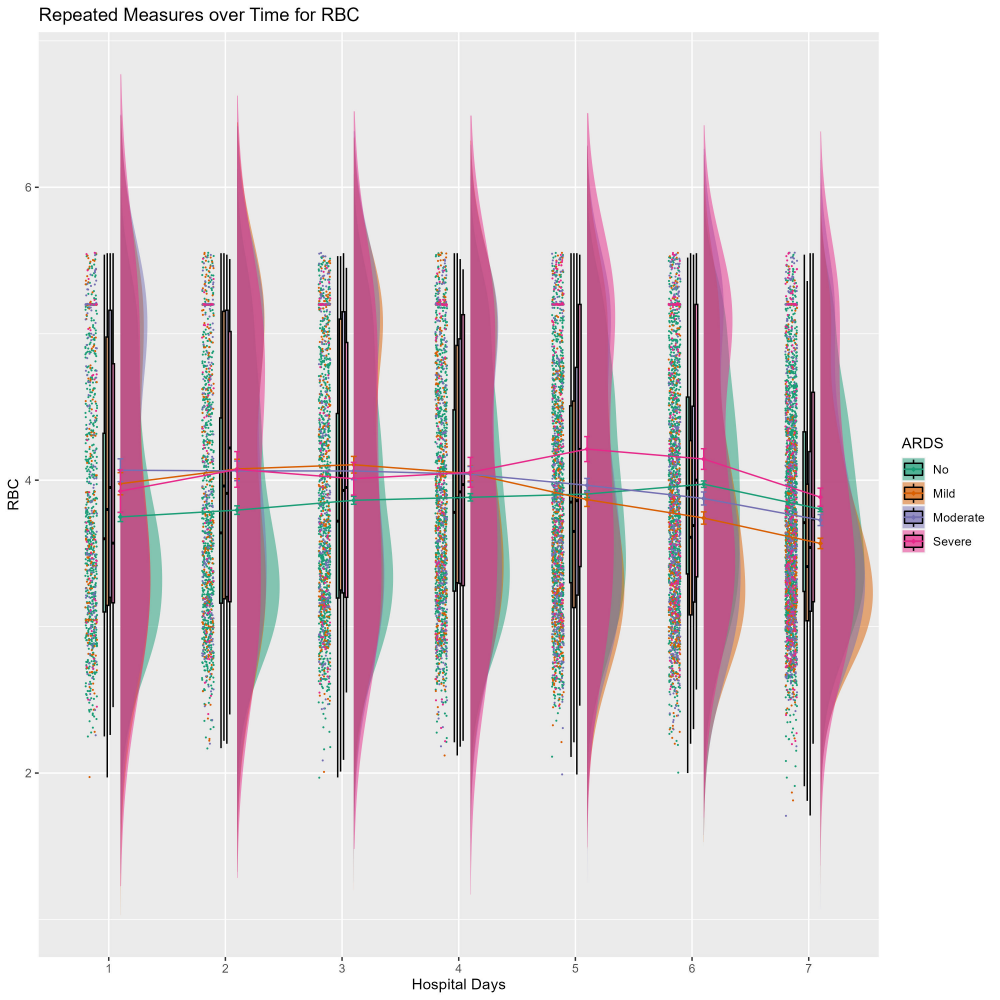


## 9) RDW


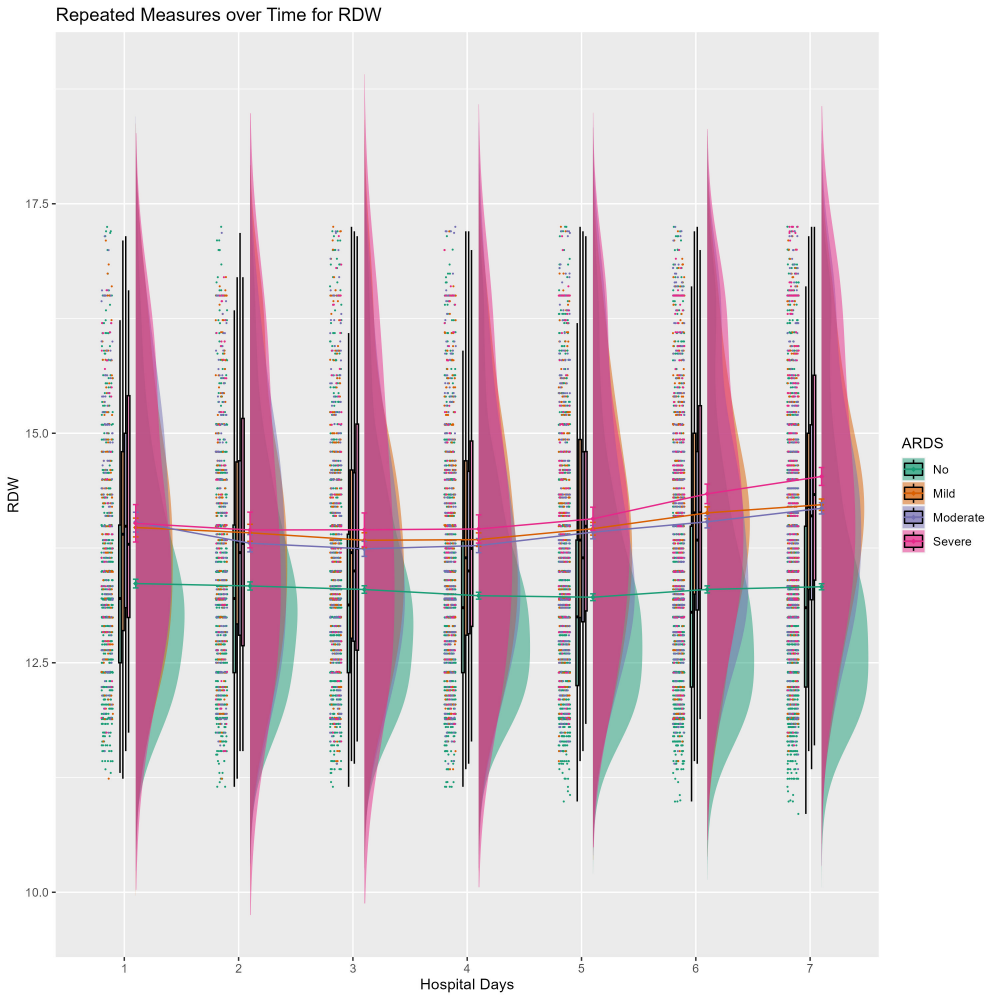


## 10) Hct


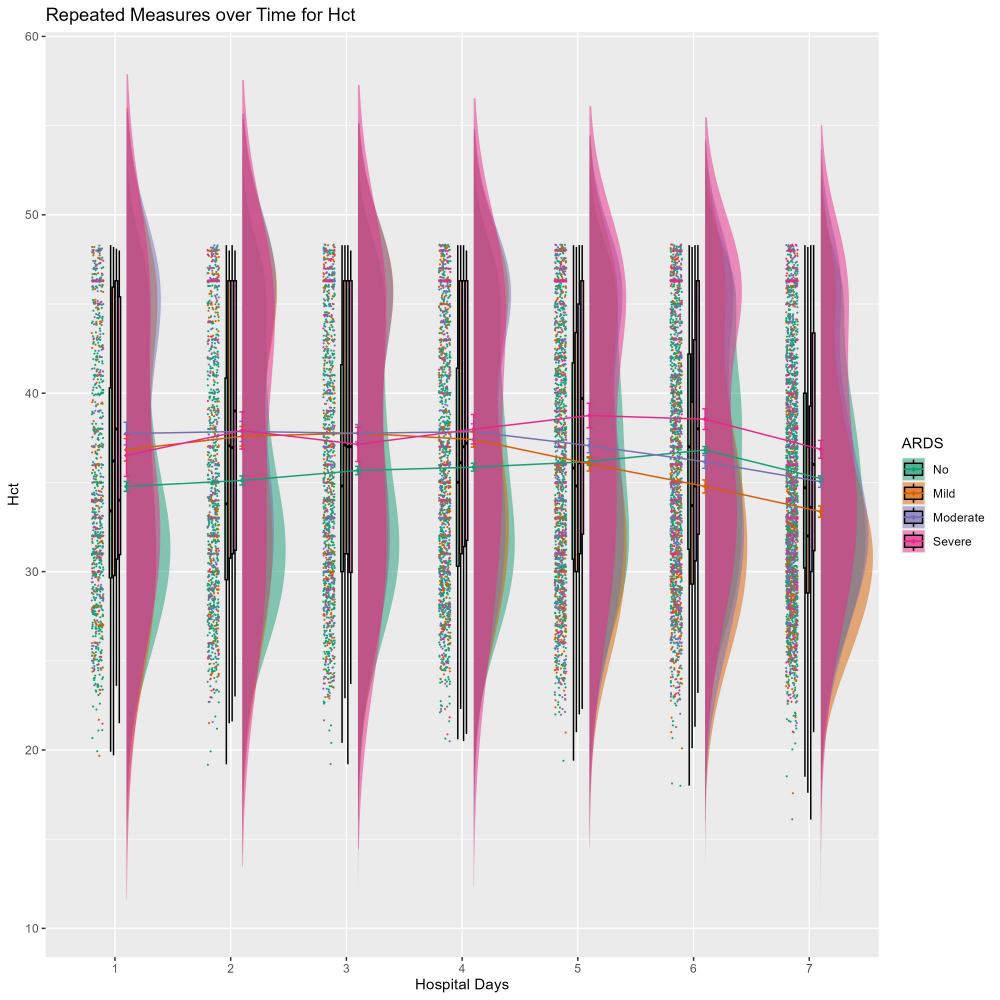


## 11) Hb


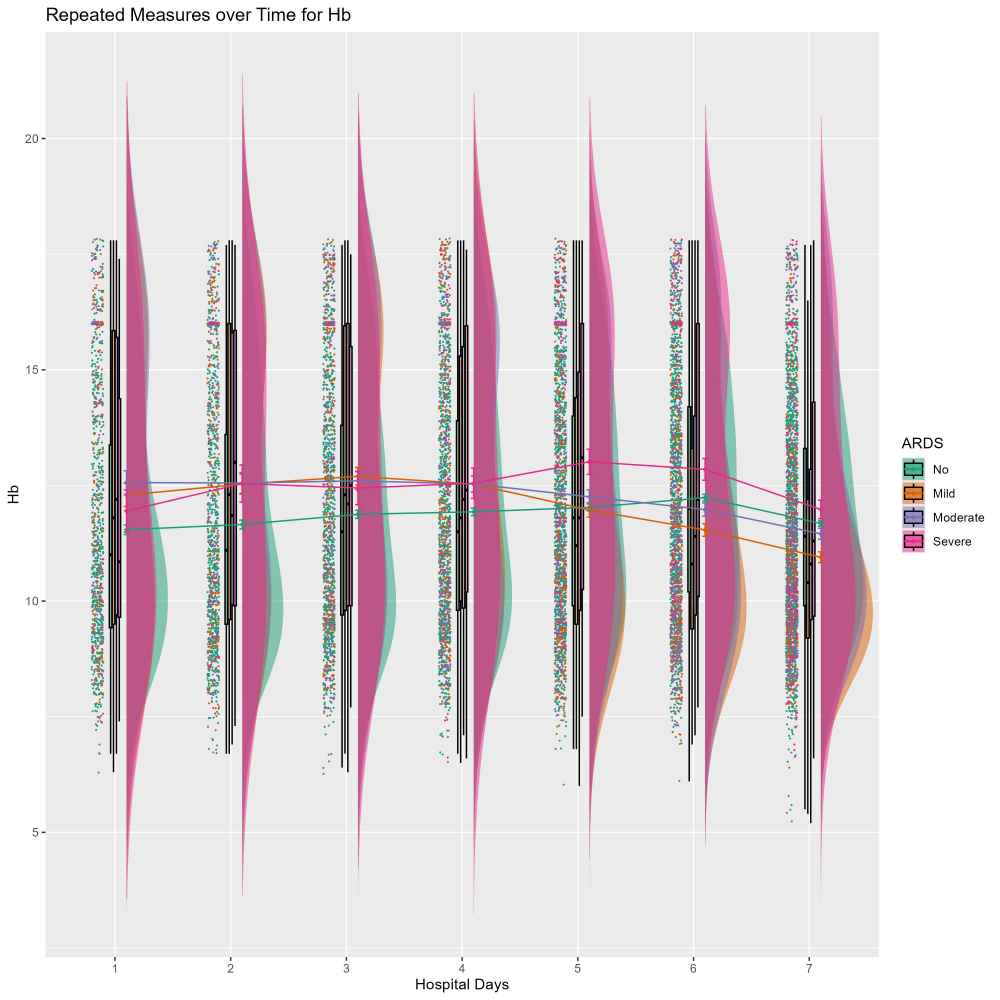


## 12 MCV


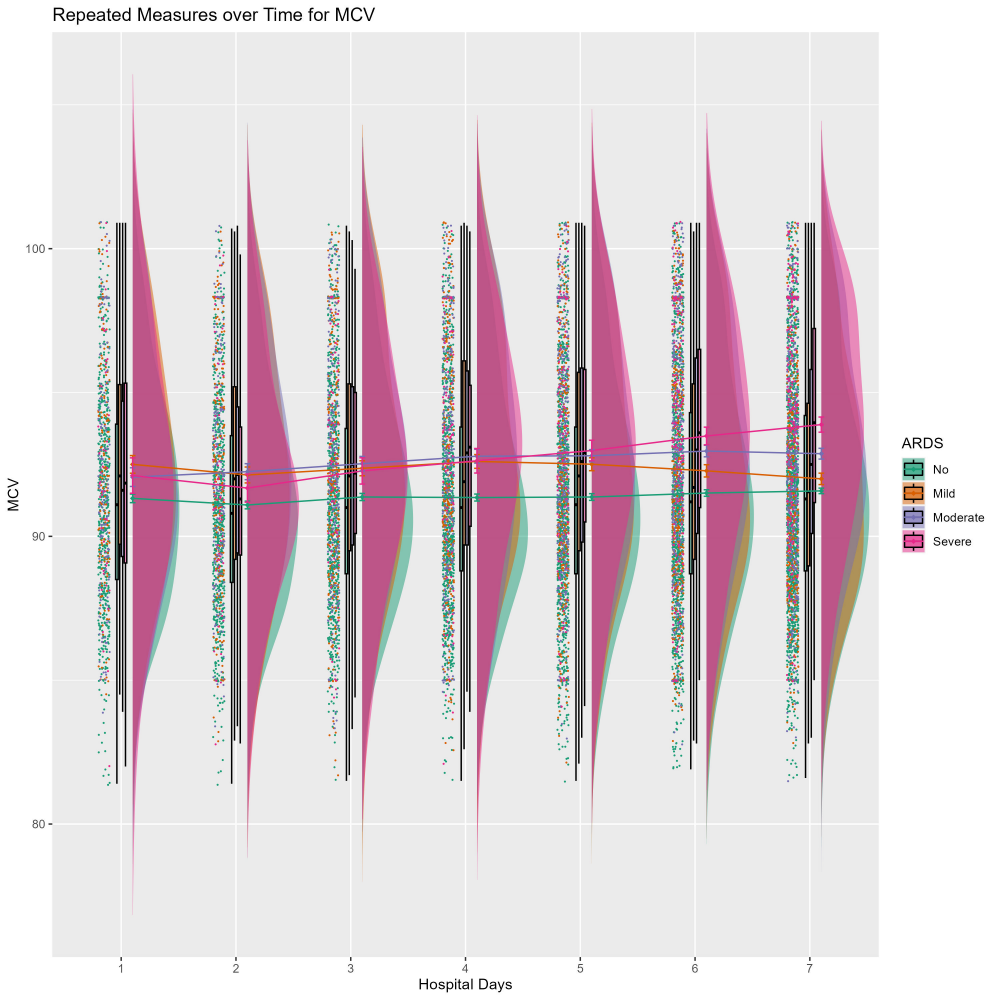


## 13) MCH


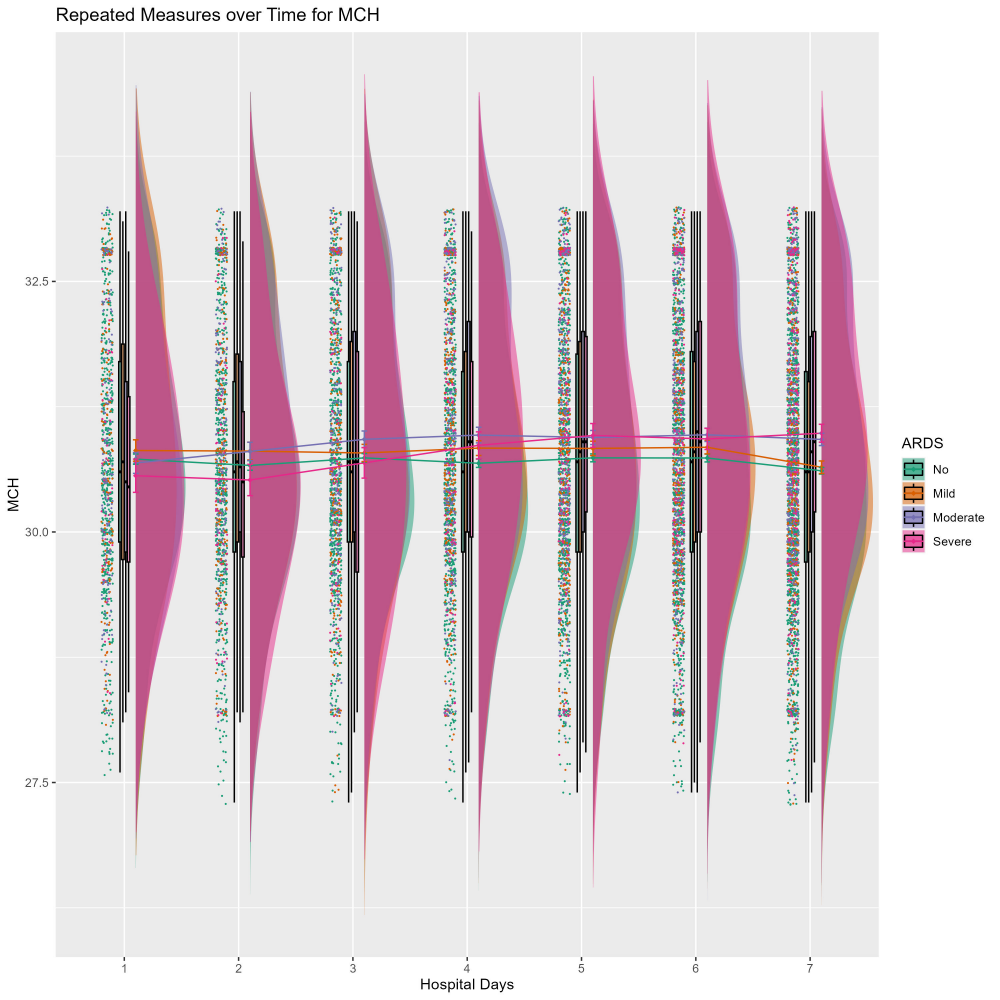


## 14) MCHC


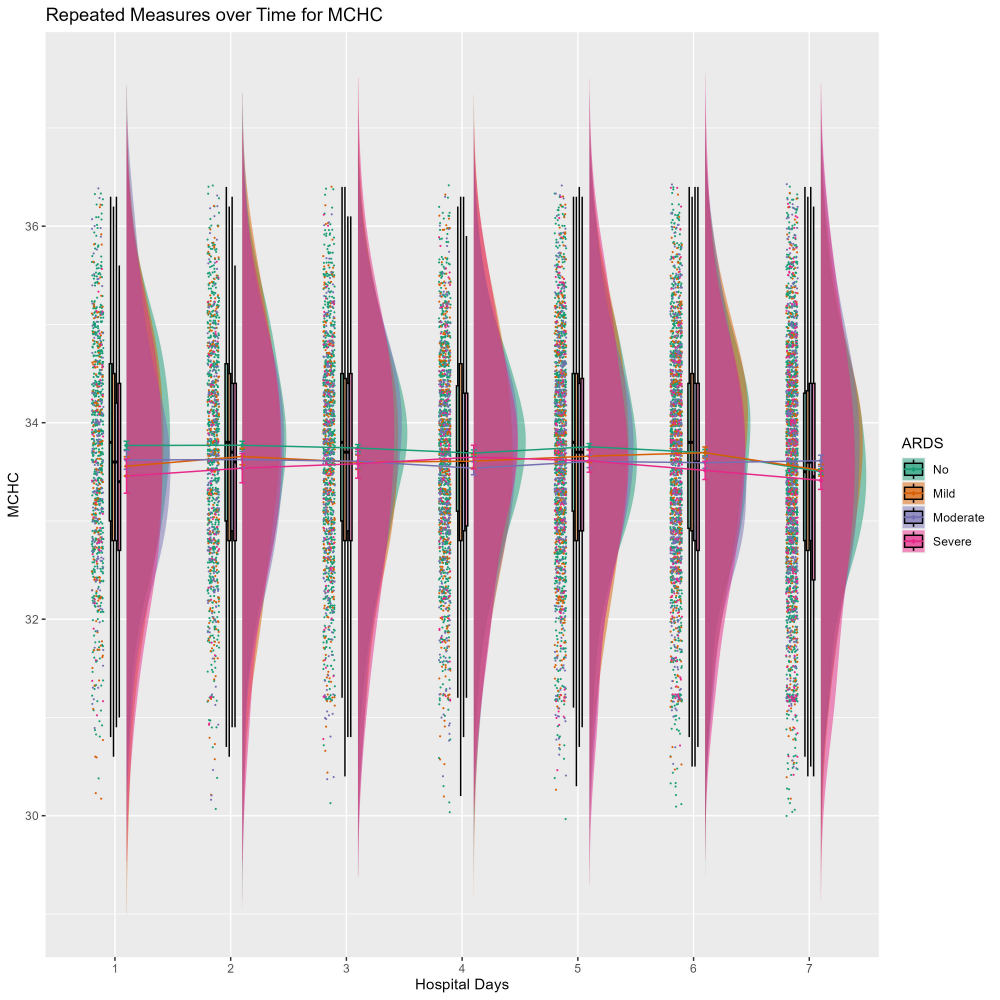


## 15) Platelet


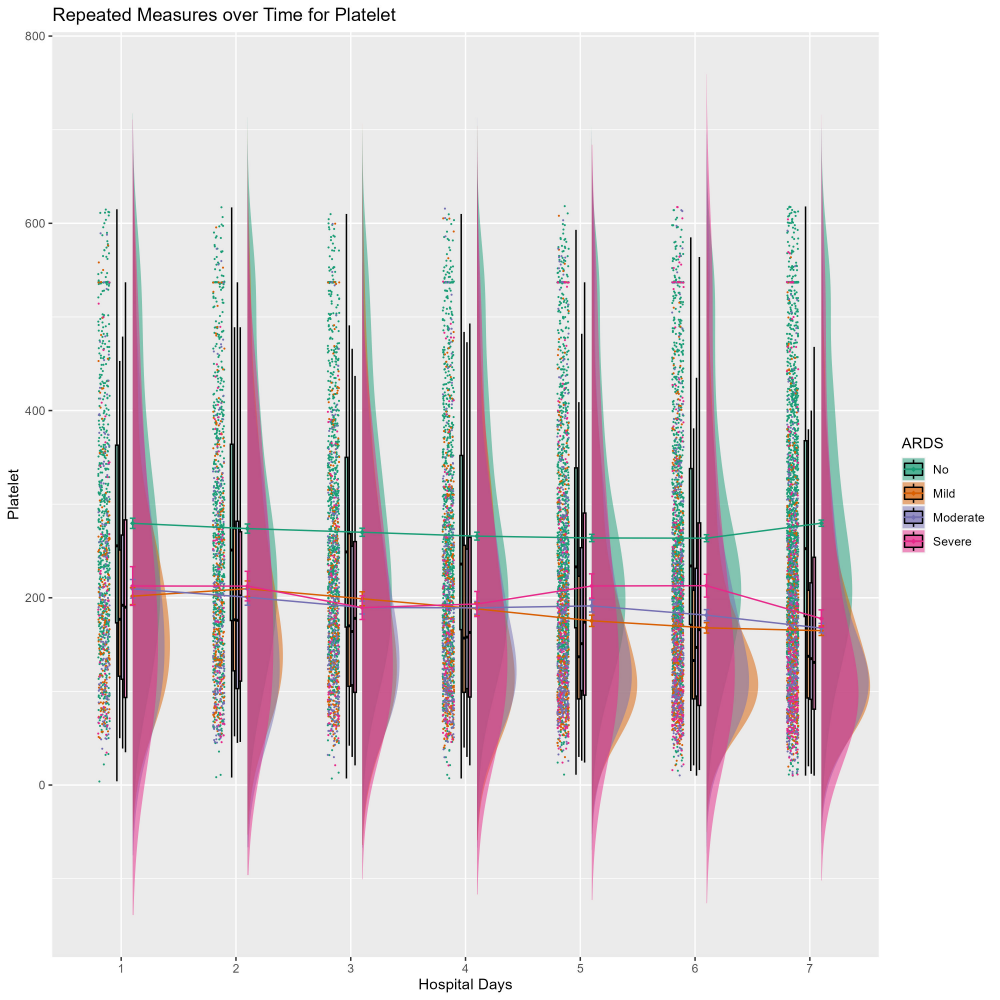


## 16) MPV


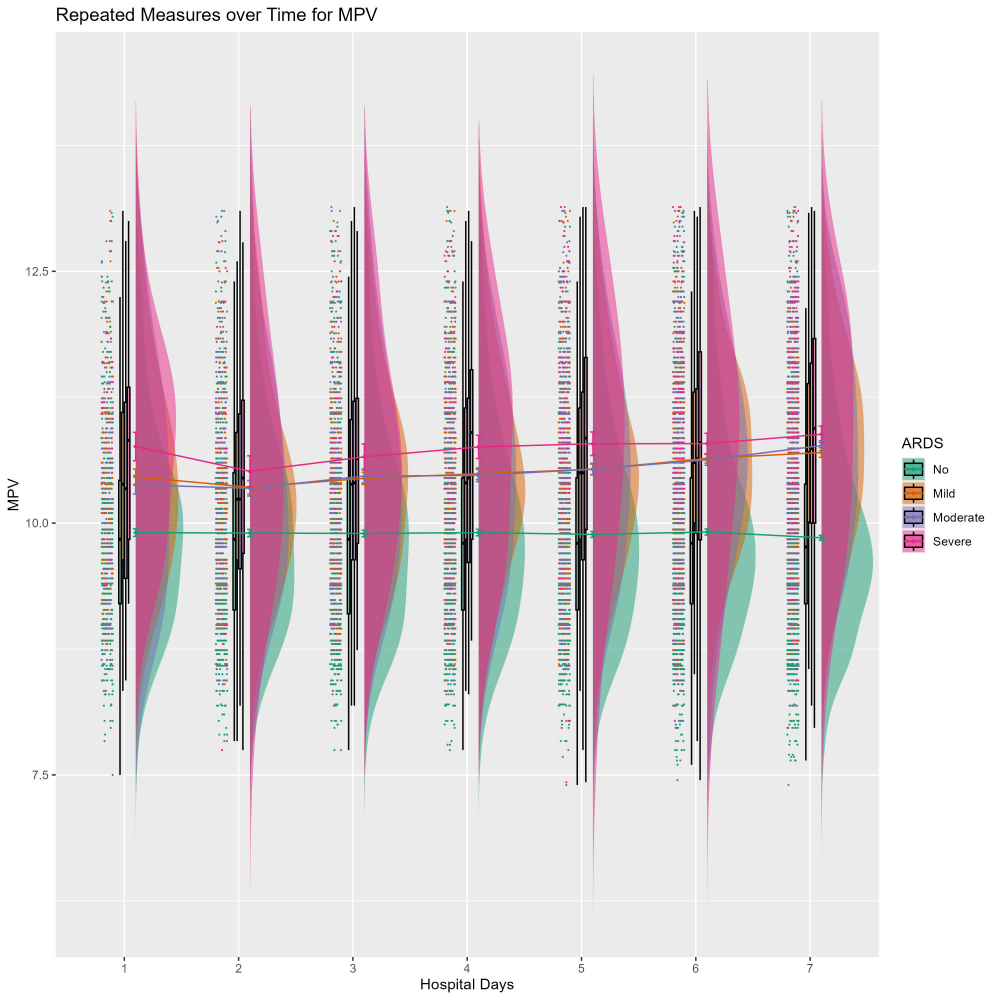


## 17) PDW


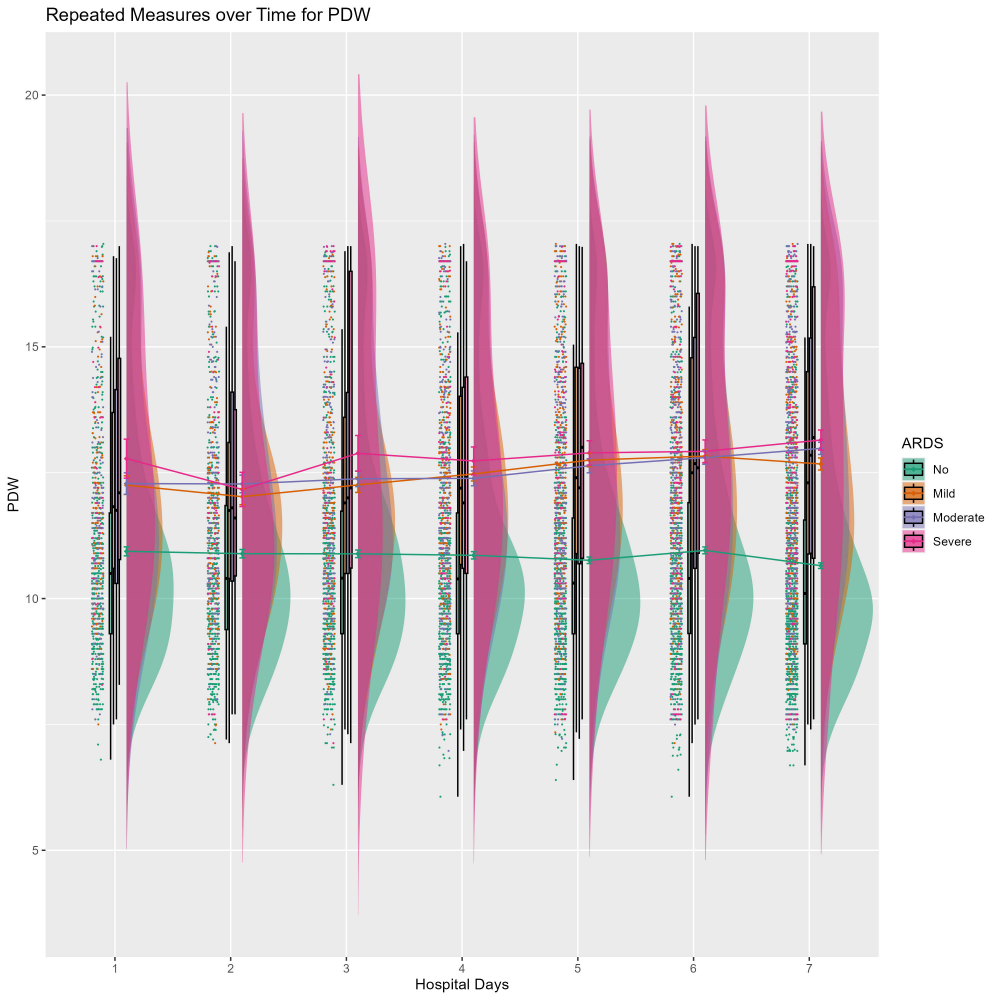


## 18) PCT


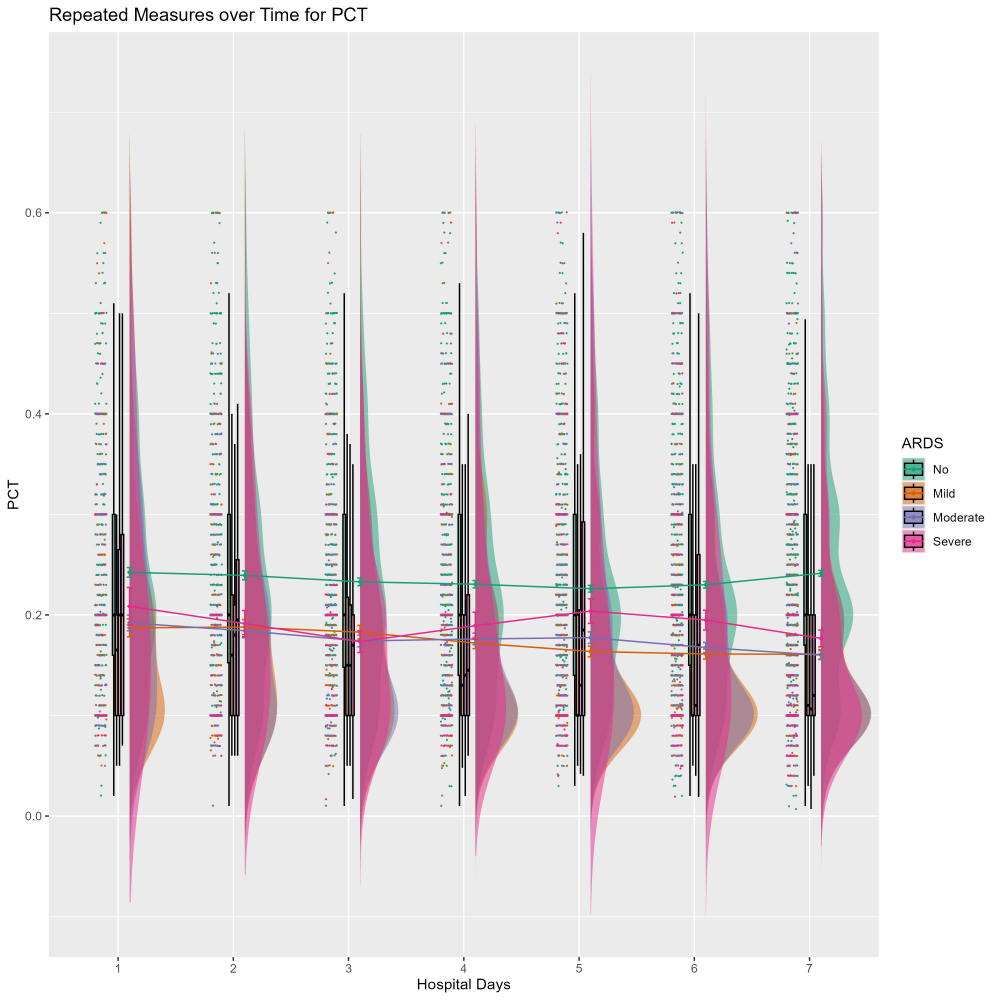


## 19) NLR


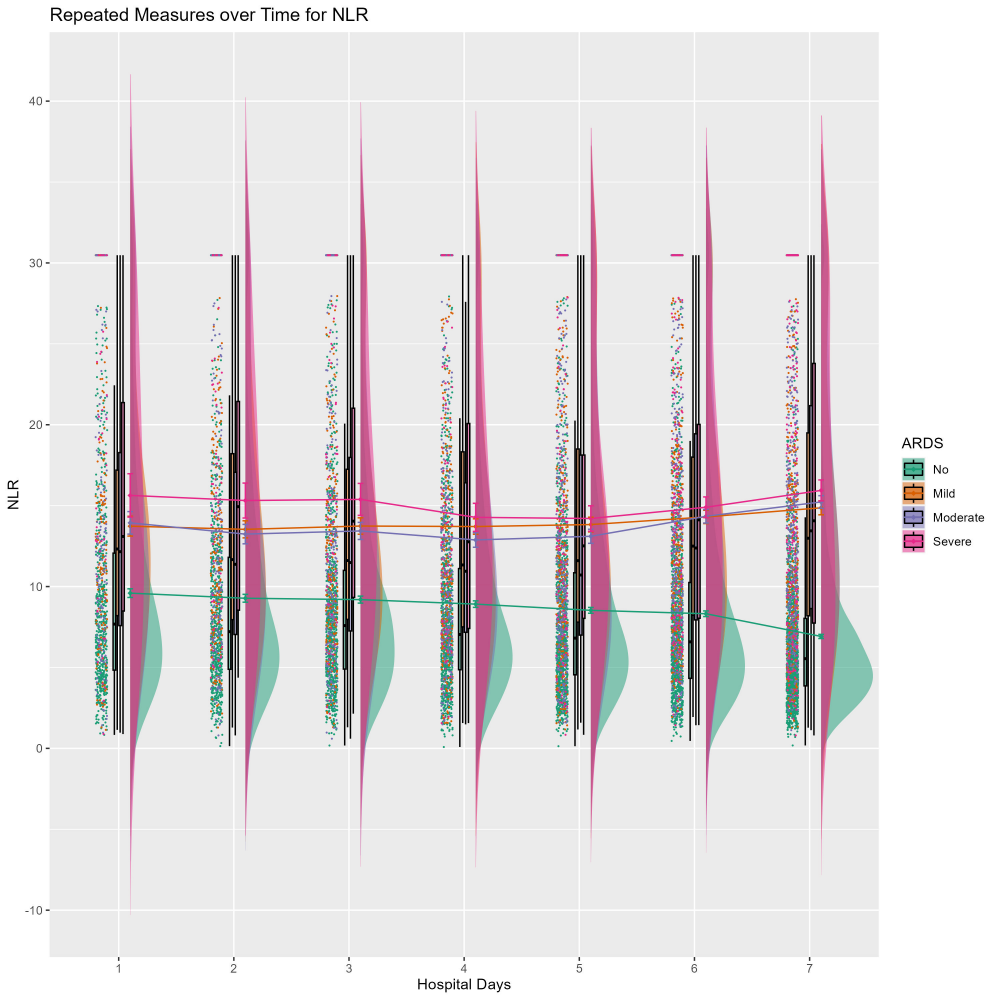


## 20 PLR


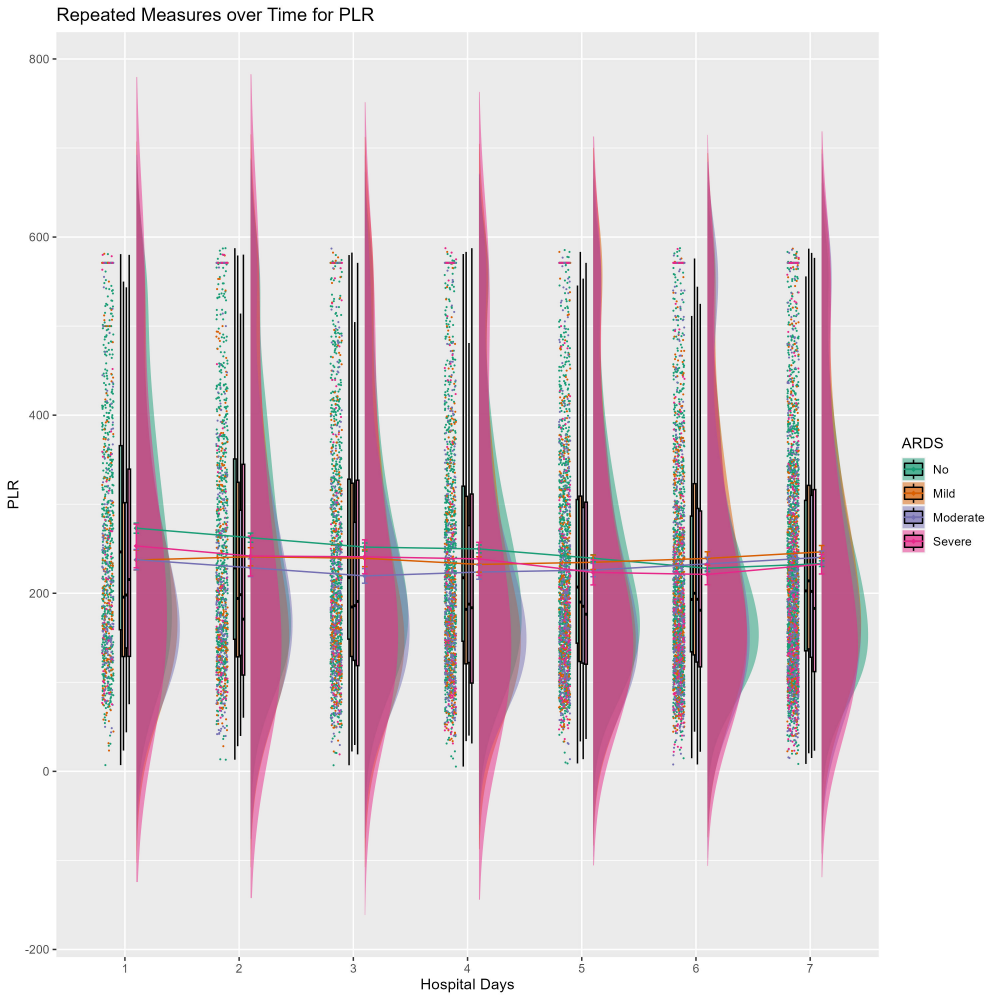


## 21 MLR


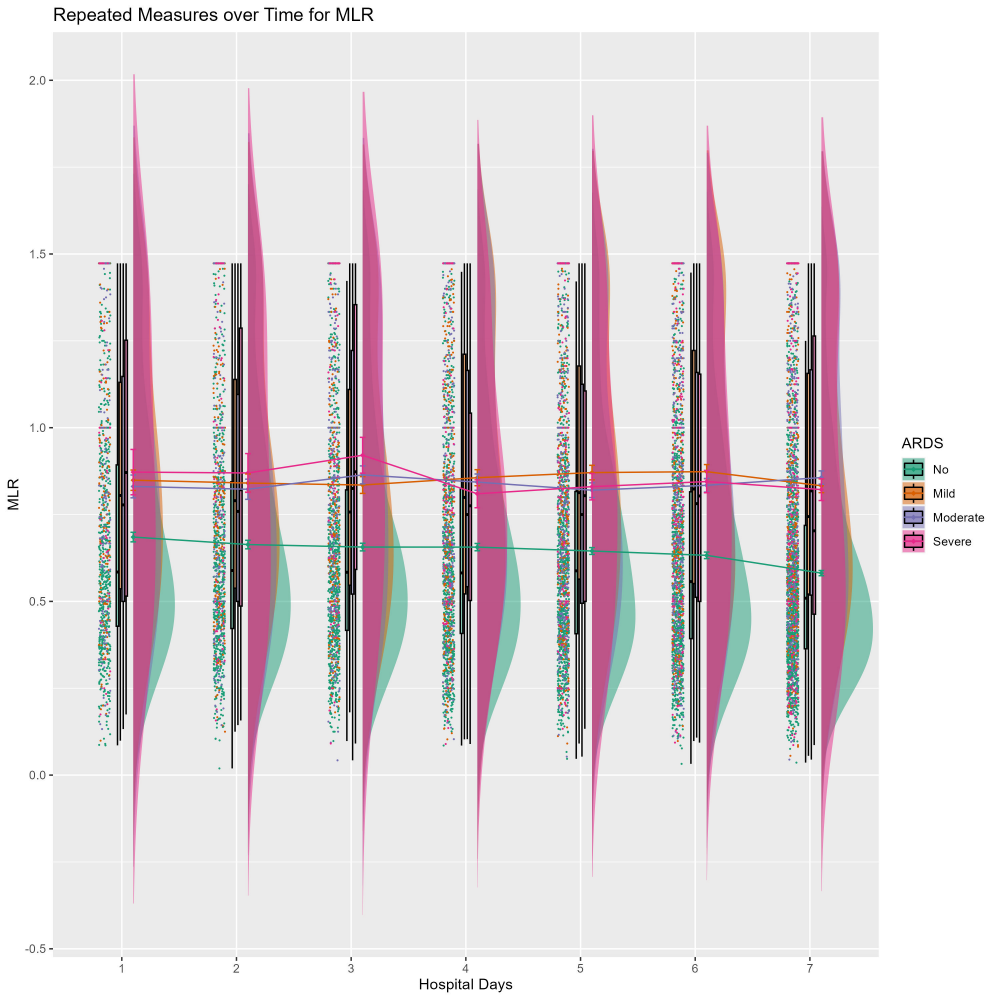


## 22) SII


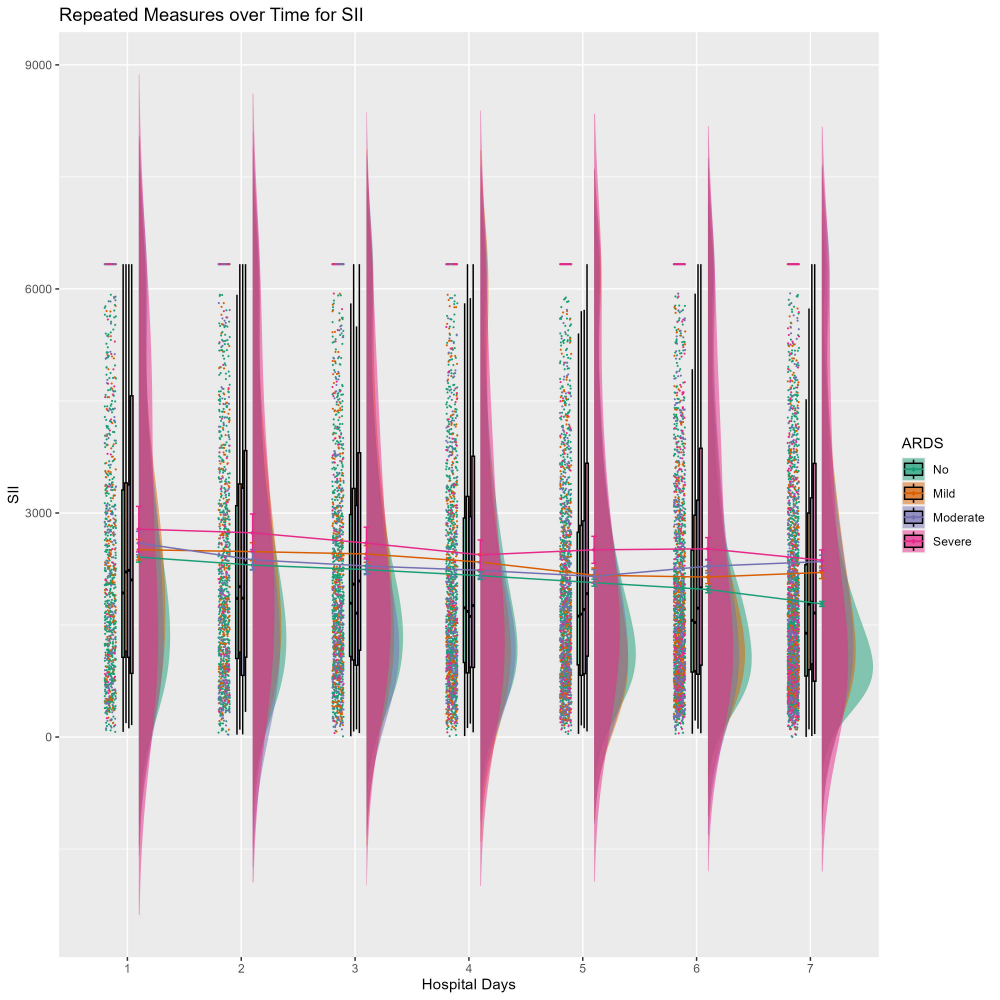


## 23) MPVPR


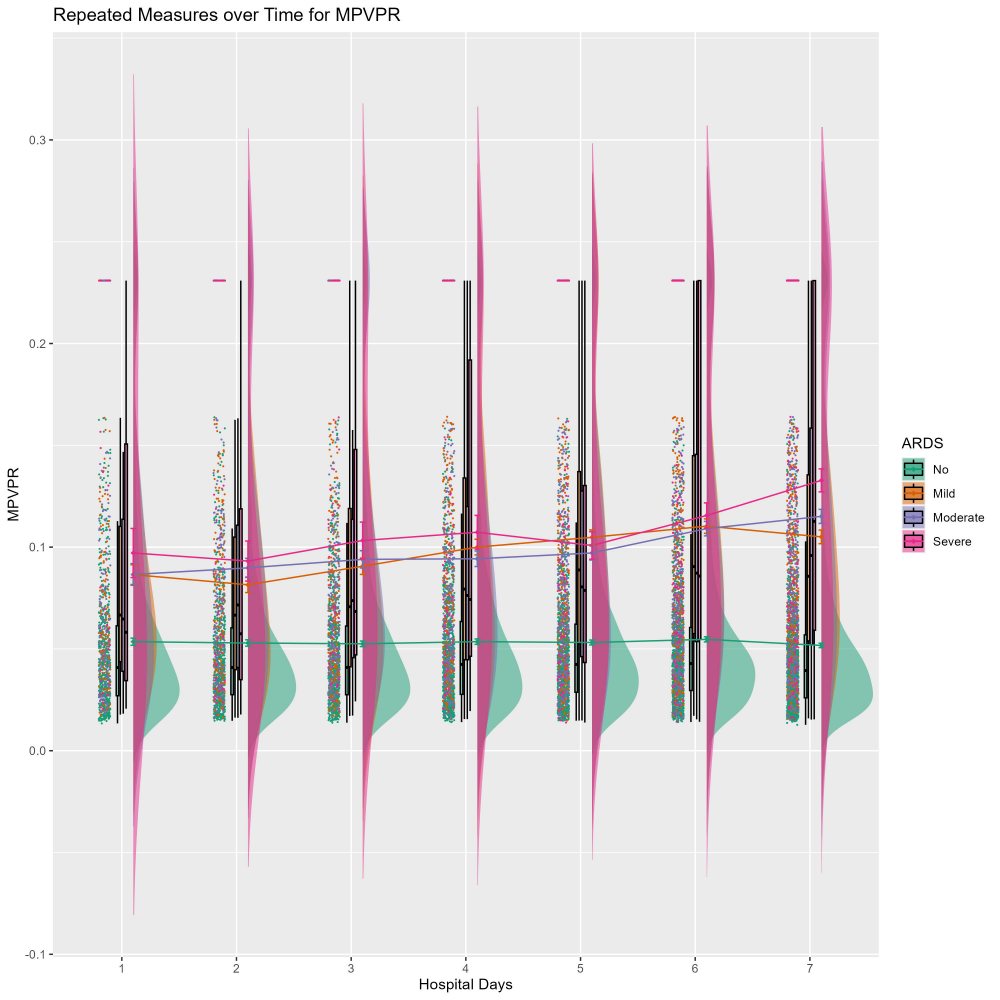


## 24) MPVLR


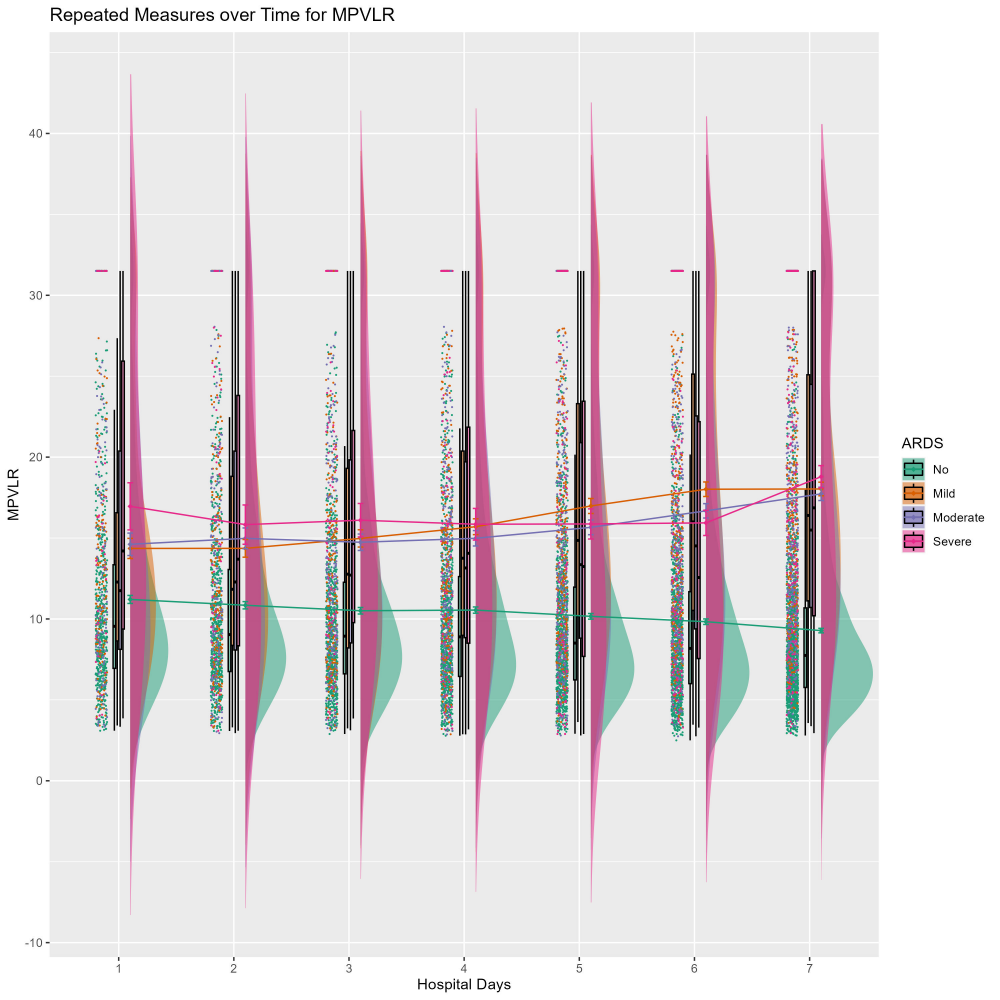


## 25) MPVMR


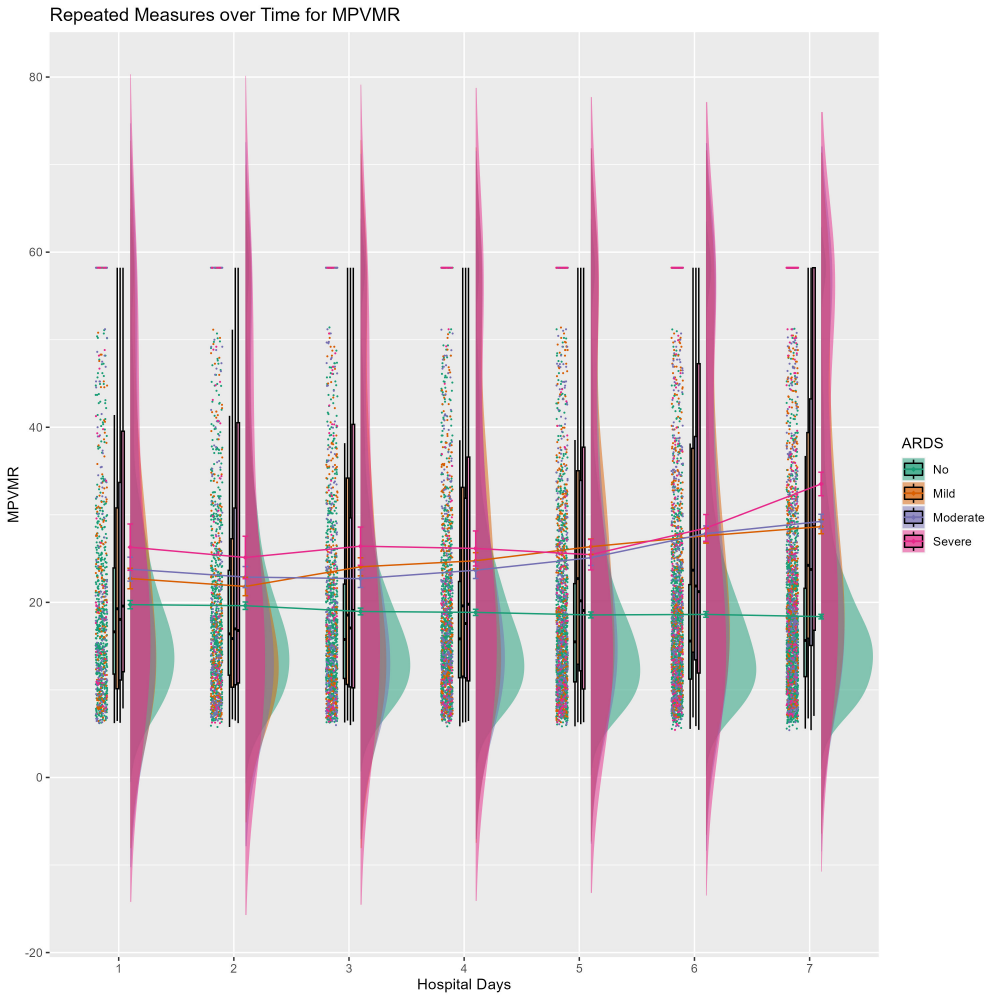


## 26) MPVNR


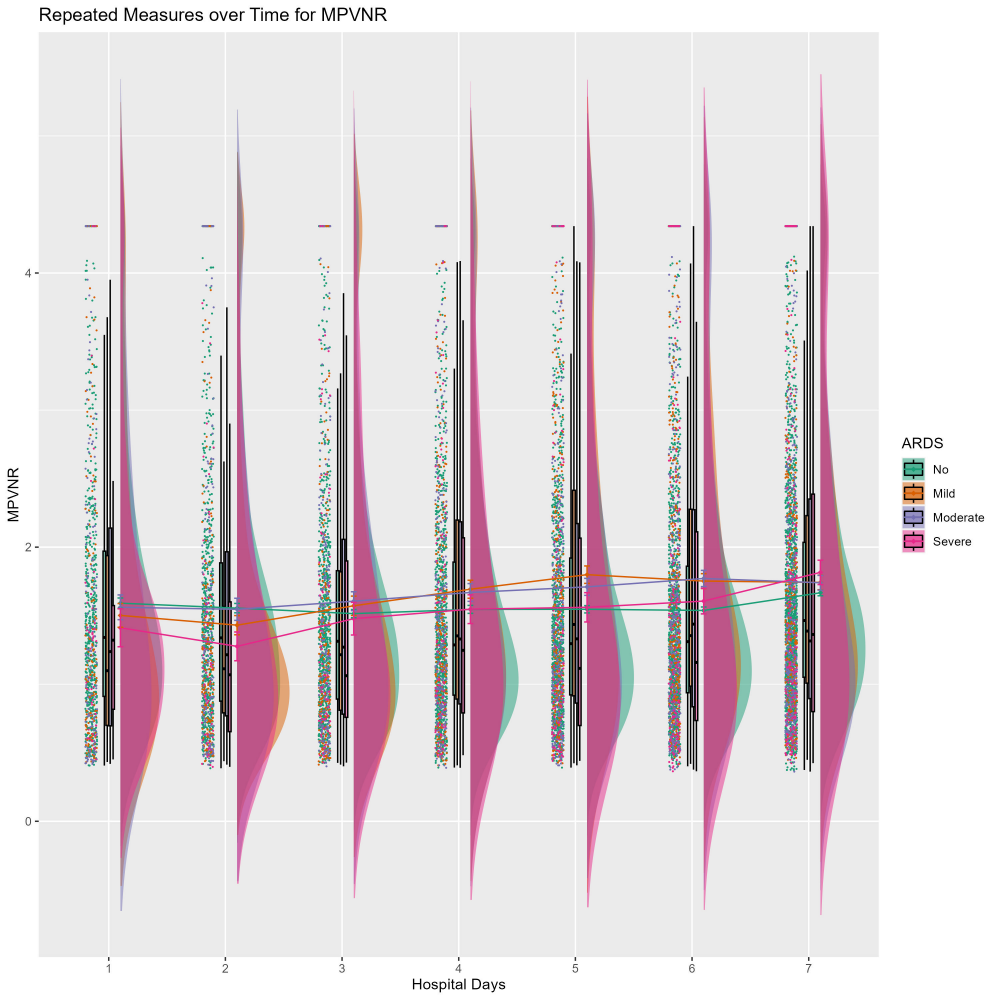

Supplement: Supplementary file 1 — Supplementary Information. [file 41598_2024_62235_MOESM1_ESM.docx]
